# Toward Animal-Free Phototoxicity Testing: A Keratinocyte-Based UVA Screening Assay to Distinguish Photoirritation from Photoallergy

**DOI:** 10.3390/toxics14080726

**Published:** 2026-08-16

**Authors:** Adriana S. Maddaleno, Elisabet Teixidó, Laia Guardia-Escoté, Montserrat Mitjans, Maria Pilar Vinardell

**Affiliations:** 1 Physiology, Department of Biochemistry and Physiology, Universitat de Barcelona, 08028 Barcelona, Spain; adrianamaddaleno@ub.edu; 2 Toxicology, Department of Pharmacology, Toxicology and Therapeutic Chemistry, Universitat de Barcelona, 08028 Barcelona, Spain; eteixido1511@ub.edu (E.T.); laia.guardia@ub.edu (L.G.-E.)

**Keywords:** phototoxicity, photoirritation, photoallergy, UV radiation, non-animal methods

## Abstract

Phototoxicity represents an increasing concern due to the presence of photoreactive chemicals in consumer products, including household products, pharmaceuticals, and cosmetic formulations. Upon exposure to solar radiation, these compounds may induce photoirritation, a non-immune-mediated inflammatory response, or photoallergy, an immune-mediated hypersensitivity reaction. However, the ability to discriminate between photoirritant and photoallergic responses remains an unmet need, as no validated in vitro assay is currently available for this purpose. Therefore, the development of reliable non-animal approaches is essential to address ethical concerns and comply with current regulatory restrictions on animal testing. Interleukin-18 (IL-18) has been proposed as a potential biomarker of allergic and photoallergic responses. In this study, we developed an in vitro assay adapted from OECD Test Guideline 432 using reference phototoxic compounds and demonstrated that exposure to the photoallergenic compound benzophenone induced IL-18 production. Furthermore, multiplex cytokine profiling identified two additional preliminary candidate biomarkers for discriminating between photoallergic and photoirritant responses. Matrix metalloproteinase-1 (MMP-1) was associated with photoallergic responses and was upregulated following exposure to chlorpromazine and benzophenone, whereas interleukin-6 (IL-6) was associated with photoirritant responses and was upregulated following exposure to 8-methoxypsoralen. Collectively, these findings provide proof-of-concept evidence supporting the future development of a novel non-animal strategy for predicting and discriminating between photoirritant and photoallergenic compounds.

## 1. Introduction

Cutaneous exposure to solar radiation can induce a broad spectrum of adverse effects such as carcinogenesis and photoaging, encompassing photosensitivity reactions. Photosensitivity is defined as an abnormal skin response to electromagnetic radiation, particularly ultraviolet (UV) light, mediated by the presence of endogenous or exogenous chromophores within the epidermis or dermis [1]. The most common causative agents are photoactive compounds found in topically applied formulations or systemically administered drugs. Phototoxicity is defined as an acute cutaneous reaction arising following the topical or systemic administration of a chemical substance upon exposure to light radiation, predominantly within the UVA spectrum, although photosensitive reactions induced by UVB, visible light, or a combination of different wavelengths have also been described [2]. In the context of topical exposure, the terms photoirritation and phototoxicity are generally considered functionally synonymous. The pathophysiological mechanisms underlying phototoxicity are multifactorial and often occur simultaneously. Following absorption of UVA radiation, a photosensitizing molecule undergoes a transition from its ground state to an electronic excited state [2]. Photoallergy is a delayed-type hypersensitivity reaction resulting from the interaction between a photoallergen and UV radiation [3]. The wavelength of incident light is a critical determinant of this process, with UVA being required for the photoactivation of most phototoxic and photoallergenic compounds. Compared with phototoxicity, photoallergic reactions generally require a lower energy threshold for induction, reflecting their immunologically mediated nature. Photoallergy elicited by exogenous agents almost invariably manifests as a T-lymphocyte–mediated delayed-type hypersensitivity (DTH) response, involving an initial sensitization phase followed by an elicitation phase upon subsequent re-exposure to the photoallergen [1]. Following photoactivation by UVA, the photoallergen reaches an electronically excited state and subsequently transfers its energy to surrounding molecules. During this process, the activated molecule forms a stable covalent bond with an endogenous carrier protein, generating an immunogenic hapten-protein conjugate, which constitutes the complete antigen responsible for initiating the adaptive immune response [4]. Topically applied agents are the most common causes of photoallergic skin reactions. Among these, sunscreen formulations containing UV filters, topical antimicrobial agents, and topical nonsteroidal anti-inflammatory drugs are the photoallergens most frequently implicated in clinical practice.

Knowledge of the pathophysiological mechanisms underlying allergic contact dermatitis at the molecular and cellular levels, together with a better understanding of the chemical characteristics of sensitizing compounds, has enabled the OECD to adopt several protocols for the identification of contact allergens [5]. Allergic contact photodermatitis can be considered as a specific form of contact hypersensitivity in which UV radiation activates the allergen, thereby triggering pathophysiological mechanisms like those involved in allergic contact dermatitis. Recently, an adverse outcome pathway (AOP) has been proposed to support the development of novel alternative methods [6]. This AOP includes four key events that provide a framework for both the development of alternatives to animal testing and the design of integrated testing strategies (ITS) for evaluating the skin photosensitisation hazard of chemicals. Based on this framework, the authors proposed a non-animal testing strategy for the identification of photoallergens. The first step involves UV/Vis (Ultraviolet/Visible) spectroscopy analysis [7] to determine the molar extinction coefficient (MEC). Chemicals with a MEC greater than 1000 L⋅mol^−1^⋅cm^−1^ [8] are subsequently evaluated using a reactive oxygen species (ROS) assay [8] to confirm their photo-reactivity. If a chemical is identified as a strong ROS generator, the next step consists of protein reactivity assays and/or cell-based tests. Protein reactivity assays evaluate the molecular initiating event by measuring the ability of chemicals to form covalent adducts with proteins or model peptides [9]. In contrast, cell-based assays assess subsequent cellular responses triggered downstream of this event, including oxidative stress, activation of intracellular signalling pathways, and the release of inflammatory mediators [5]. As these processes involve multiple cell types and mechanisms, different assay formats may be used, with varying degrees of biological complexity, from single-cell cultures to advanced co-culture and reconstructed tissue models. Therefore, these approaches provide complementary rather than equivalent mechanistic information. The work of de Àvila et al. also highlighted the urgent need to develop new approach methodologies (NAMs) and corresponding risk assessment strategies to ensure the safety evaluation of chemical-induced photoallergy [6]. In a recent review, we examined the current landscape of alternative methods for detecting photosensitization, with a focus on recent literature and an evaluation of the fundamental principles underlying the various proposed assays [10]. Considering all these aspects, the aim of the present work is to investigate whether a human keratinocyte-based in vitro model could identify candidate biomarkers associated with the early key events of photoallergy, as an initial step toward the development of a more human-relevant in vitro assay to distinguish photoirritation from photoallergy.

## 2. Materials and Methods

### 2.1. Cells and Cell Maintenance

The human keratinocyte cell line HaCaT (Eucellbank, Celltec UB, Universitat de Barcelona, Barcelona, Spain) [11] was chosen for the study. Cell growth and maintenance of HaCaT cells were performed in 75 cm^2^ flasks containing Dulbecco’s Modified Eagle’s Medium (DMEM, Cytiva, Marlborough, MA, USA) supplemented with 10% heat-inactivated fetal bovine serum (FBS, Cytiva, Marlborough, MA, USA), 2 mM L-glutamine and 100 U/mL:100 U/mL streptomycin-penicillin mixture (Corning, New York, NY, USA) (10% FBS-DMEM) at 37 °C in a 5% carbon dioxide humidified incubator. Cell growth and morphology were continuously monitored throughout the experiments, and cell subculture and seeding were performed when cells reached nearly 80% confluence after detaching cells with Trypsin-EDTA (Cytiva, Marlborough, MA, USA).

### 2.2. Establishment of Experimental Conditions

The phototoxicity assay was performed following test guideline OECD TG 432 [12] with some modifications. Briefly, the murine Balb/c 3T3 cell line recommended in the guideline was replaced with the human keratinocyte line HaCaT. Cells were seeded at a density of 1 × 10^5^ cells/mL in 96-well plates, excluding the outer wells and maintained for 24 h at 37 °C, 5% CO_2_ and 92% humidity prior to being exposed to the chemicals. To establish the experimental condition of UVA irradiation, we selected chlorpromazine hydrochloride (CPZ; Sigma, Aldrich, Steinheim, Germany) and sodium lauryl sulphate (SLS, Sigma), the reference chemicals recommended in OECD TG 432 [12] (Table 1), which were used as positive and negative controls, respectively. For each chemical, two plates were prepared, one exposed to UVA radiation and the other kept in the dark. On the following day, fresh serial dilutions of each compound were prepared in sterile phosphate buffer solution (PBS). After removing the culture medium, cells were incubated with the test solutions for 1 h before either UVA irradiation or dark incubation. Following treatment, the solutions were removed, fresh culture medium was added, and cells were incubated for an additional 24 h. Cell viability was determined 24 h later, and the IC_50_ (concentration that induces 50% mortality) was calculated as described in Section 2.4 to assess the phototoxic potential of the tested compounds. Untreated cells exposed only to PBS served as viability controls, and all concentrations were tested in triplicate in each independent experiment.

### 2.3. Irradiation Conditions

UVA irradiation was performed in a cabinet equipped with three Actinic BL UVA 15W/10 FAM/10X25BOX (15 W, Philips, Amsterdam, The Netherlands). The lamps have a dominant emission peak at approximately 365 nm with most of the emitted radiation within the 350–370 nm region and less than 0.1% UVB emission. Before each experiment, the lamps were switched on for 10 min to allow stabilization, and the UVA irradiance was measured using a calibrated radiometer (HD2302, Delta OHM, Selvazzano Dentro, Italy) fitted with a UVA probe sensitive over the 315–400 nm range (peak spectral response ~360 nm) [12]. The probe was positioned horizontally at the same distance from the lamps as the cell monolayer to accurately determine the irradiance reaching the cultures. Irradiation was conducted at room temperature with culture plates placed at a fixed distance of 10 cm from the light source with the culture plate lid removed. Temperature inside the cabinet did not exceed 30 °C in any experiment and was monitored before and immediately after UVA exposure using a calibrated thermometer. The exposure time required to deliver the desired UVA dose was calculated according to the following equation:

UVA Dose (J/cm^2^) = Irradiance (W/cm^2^) × Exposure time (s), (1)

Thus, based on the lamps’ irradiance (2100–2300 µW/cm^2^), cells were exposed to UVA light for a maximum of 40 min to deliver a total UVA dose of 5 J/cm^2^. During UVA exposure, the non-irradiated control plate was maintained in the dark under the same temperature and environmental conditions as the irradiated plate.

### 2.4. Determination of Cell Viability

Cell viability was assessed approximately 24 h after irradiation using the two assays in parallel: the neutral red uptake (NRU) assay, as previously described [12], and the MTT assay following the protocol established by our research group [13].

Briefly, the culture medium was discarded, and cells were treated with 100 µL of either 0.05 mg/mL NR solution or 0.5 mg/mL MTT, both prepared in serum-free DMEM, for almost 3 h (37 °C, 5% CO_2_ and 92% humidity). The supernatant was then removed by decanting the plates, followed by the addition of destain solution (NRU assay) or DMSO (MTT assay). Plates were gently shaken for 10 min to ensure complete dye solubilization. Finally, absorbance was determined at 550 nm (Tecan Sunrise^®^ microplate reader, Männedorf, Switzerland).

Then, cell viability was calculated considering that the absorbance of untreated cells corresponds to 100% of cell viability. The establishment of the UVA dose was based on the lack of deleterious effects reported for the irradiated untreated cells (cell viability ≥80%) and the potential phototoxicity associated with CPZ and SLS correctly predicted by the Photo Irritation Factor (PIF) and/or the Mean Photo Effect (MPE) [12].

PIF is a numerical parameter used to assess phototoxicity by comparing the cytotoxicity of a chemical in the presence and absence of UVA/visible irradiation based on IC_50_ values, whereas MPE evaluates the overall difference between the cell viability concentration-response curves with and without exposure to UVA/visible light. According to OECD 432 [12], a chemical is classified as phototoxic when the PIF is ≥5 and/or MPE is ≥0.15; as non-phototoxic when PIF is <2 and/or MPE is <0.1, and as equivocal when values fall between these thresholds.

### 2.5. Further Chemicals Assayed

Once the irradiation conditions were established, three more chemicals based on their phototoxic or non-phototoxic characteristics were assessed (Table 1). For each experiment, fresh stock solutions were prepared in sterile PBS for *p*-phenylendiamine or in DMSO for the poorly water-soluble chemicals Benzophenone and 8-Methoxypsoralen, with the final maximal DMSO concentration not exceeding 0.2%. Then, the PIF was calculated from the IC_50_ values obtained under dark and UVA irradiation conditions, whereas the MPE was calculated from the corresponding concentration–response curves.

Test chemicals were selected to represent different adverse reaction profiles relevant to photosafety assessment, including photoirritants (8-MOP), photoallergens (benzophenone), compounds exhibiting both photoirritant and photoallergic properties (CPZ), contact allergens without established phototoxicity (PPD), and irritants without phototoxic properties (SLS). The classification assigned to each compound was based on its predominant adverse reaction reported in the literature. Accordingly, CPZ was selected as a representative compound exhibiting both photoirritant and photoallergic properties, as it is one of the best-characterized chemicals known to induce both types of photosensitivity reactions [14,15,16]. BZ was selected as a representative photoallergen because it is widely recognized as a photoallergic compound in both clinical and experimental studies [14,17,18]. 8-MOP was selected as a reference photoirritant because it is widely recognized as a phototoxic/photoirritant compound in both experimental and clinical literature [14,16,19,20]. Although several reports have described photoallergic reactions to 8-MOP [21], these are relatively rare and mainly based on case reports or small clinical series [20]. Therefore, it was considered representative of a predominantly photoirritant compound for the purpose of this study. PPD was intentionally included as a representative contact allergen [14,16] without established intrinsic phototoxicity to evaluate whether the proposed biomarkers specifically respond to photoallergic mechanisms rather than to contact allergenicity itself. Finally, SLS was included as a representative non-phototoxic irritant, since it induces irritation through non-immunological mechanisms and is commonly used as a reference irritant in toxicological studies [12,22].

**Table 1:** Chemicals studied and classification according to their phototoxicity adverse reactions.

Chemical	Abbreviation	CAS Number	Classification	Reference
Chlorpromazine HCL	CPZ	69-09-0	Phototoxic (photoirritant and photoallergen)	[14,15,16]
Benzophenone	BZ	119-61-9	Phototoxic (photoallergen)	[14,17,18]
8-methoxypsoralen	8-MOP	298-81-7	Phototoxic (mainly photoirritant)	[14,16,19,20]
*p*-phenylendiamine	PPD	106-50-3	Non-phototoxic (allergen)	[14,16]
Sodium lauryl sulphate	SLS	151-21-3	Non-phototoxic (irritant)	[12,22]

Finally, for each chemical, three concentrations were selected for the following studies. The concentrations were chosen according to their phototoxic behaviour and to ensure that minimal cell viability was ≥75–80%.

### 2.6. Determination of IL-18 Production

HaCaT cells were seeded at a density of 1 × 10^5^ cells/mL in DMEM supplemented with 10% FBS, 1% glutamine, and 1% streptomycin–penicillin and incubated for 24 h at 37 °C in a humidified atmosphere containing 5% CO_2_. Cells were then treated for 1 h with each compound in triplicate at the following concentrations: 0.125, 0.25, and 0.5 µg/mL CPZ; 0.005, 0.01, and 0.02 µg/mL 8-MOP; 1.25, 2.5, and 5 µg/mL BZ and 10, 20, and 30 µg/mL PPD. Soluble compounds were prepared directly in PBS, whereas poorly soluble or insoluble compounds were initially dissolved in DMSO and subsequently diluted in PBS to obtain the final treatment solutions. The final DMSO concentration in the wells did not exceed 0.2%, and vehicle-matched controls were included where applicable. Following treatment, cells were exposed to UVA irradiation at a dose of 4 J/cm^2^. After irradiation (30 min approximately), the treatment solutions were discarded, and fresh culture medium was added. After 24 h of incubation, cells were lysed using 0.08% Triton X-100 in PBS on ice, collected and frozen at −80 °C until subsequent analysis.

Cell lysates were subsequently used to quantify intracellular IL-18 using a commercial ELISA kit assay following the instructions of the supplier and with a detection range of 31.25–2000 pg/mL (Elabscience Biotechnology Inc., Wuhan, China). Total protein content of cell lysates was quantified using the Bradford assay. Briefly, 10 µL of cell lysate was mixed with 10 µL of formic acid and 200 µL of Bio-Rad Bradford reagent (diluted 1:4 in distilled water). A bovine serum albumin (BSA) standard curve was used for protein quantification. The absorbance was read at a wavelength of 595 nm (Tecan Sunrise^®^ microplate reader, Männedorf, Switzerland).

### 2.7. Human Inflammation and Matrix Metalloproteinase Antibody Arrays

The relative expression of inflammatory mediators and matrix metalloproteinases (MMPs) in cell culture supernatants was analyzed using the Human Inflammation Antibody Array C3 (AAH-INF-3) and Human Matrix Metalloproteinase Antibody Array C1 (AAH-MMP-1) from RayBiotech (Peachtree Corners, GA, USA). These arrays enable the simultaneous semi-quantitative detection of multiple cytokines and MMPs through capturing antibodies immobilized on nitrocellulose membranes. The concentrations used for each compound were as follows: 0.5 µg/mL CPZ, 0.02 µg/mL 8-MOP, 5 µg/mL BZ, and 10 µg/mL PPD.

All procedures were performed according to the manufacturer’s instructions. Briefly, membranes were blocked with 2 mL of the supplied blocking buffer for 30 min at room temperature. Subsequently, membranes were incubated overnight at 4 °C with 1 mL of cell culture supernatant per membrane under gentle agitation. After extensive washing, membranes were incubated with 1 mL of a cocktail of biotinylated detection antibodies, followed by washing and incubation with 2 mL of horseradish peroxidase (HRP)-conjugated streptavidin. Signal detection was performed using enhanced chemiluminescence (ECL) reagents.

Chemiluminescent signals were quantified by densitometric analysis using an Amersham Imager 680 UV with an Amersham ImageQuant TL analysis software (IQTL Ver. 10). Spot intensities were background-corrected and normalized to the positive control spots present on each membrane, following the manufacturer’s recommendations. Data were expressed as normalized relative intensity.

### 2.8. Data Analysis

For phototoxicity studies and ELISA assays, at least four independent biological experiments were performed on different days, using different cell passages and with fresh preparation of the test solutions. Each experimental condition was tested in triplicate (technical replicates). IC_50_, PIF, and MPE values were derived from absorbance data obtained using the MTT assay and Neutral Red Uptake assay and calculated with the OECD Phototox software [12]. Data were expressed as mean ± standard error of the mean (SEM) of the independent biological experiments.

Statistical significance between treatment groups for all experiments was determined using a two-way ANOVA in which concentration, irradiation condition, and the concentration × irradiation interaction were evaluated. When a significant effect of concentration was detected, Dunnett’s multiple-comparison test was used to compare each concentration with the corresponding control under the same irradiation condition (IBM SPSS statistics^®^ program v. 27, SPSS Inc., Chicago, IL, USA). Differences were considered statistically significant when *p* < 0.05, *p* < 0.01 or *p* < 0.005 as indicated in Figures and Tables.

## 3. Results

### 3.1. Establishment of UVA Dose

As a starting point, the sensitivity of HaCaT cells to UVA light was previously determined by exposing the cells to 5 J/cm^2^ (between 36 and 40 min.) and 4 J/cm^2^ (between 28 and 32 min.) of UVA light and comparing their viability to non-irradiated cells. Table 2 shows the percentage of cell viability obtained with the NRU and MTT assays. In both conditions, untreated cells exhibited acceptable cell viability when calculated by the NRU assay, whereas this was not the case with MTT at 5 J/cm^2^. Moreover, the standard errors were lower at the lower UVA dose. For this reason, further experiments were performed at 4 J/cm^2^. Nevertheless, in each experiment, cellular sensitivity to light was monitored by comparing the viability of irradiated cells with that of cells maintained in dark conditions.

**Figure 1:**
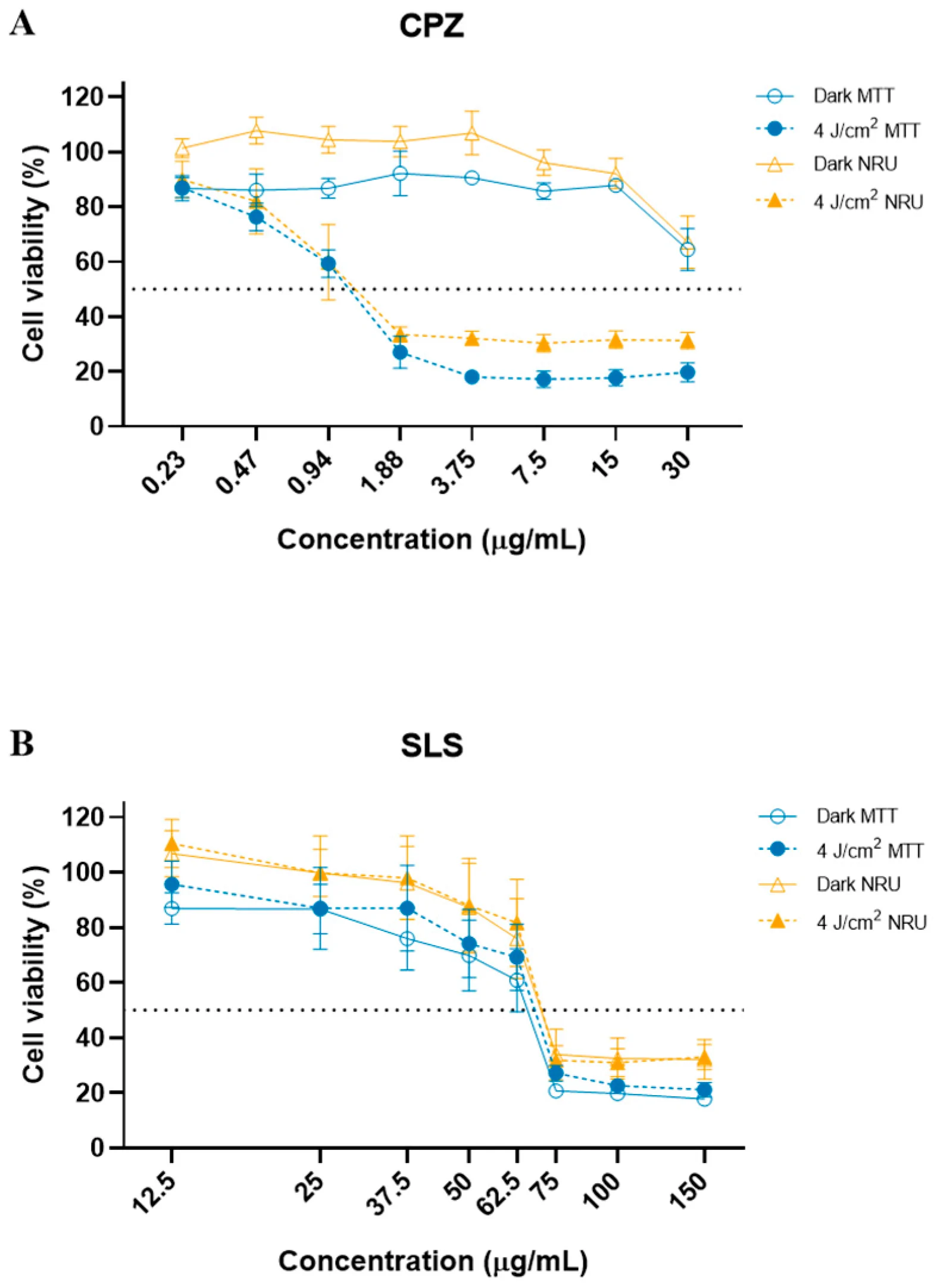
Cell viability of Chlorpromazine (CPZ, (**A**)) and Sodium lauryl sulphate (SLS, (**B**)) in dark and irradiated conditions (4 J/cm^2^ of UVA light) obtained by the NRU and MTT tests. Results are expressed as mean ± SEM of 4–5 independent biological experiments.

Once the light dose was established, the phototoxic behaviour of CPZ and SLS was assayed (Figure 1). As observed, when cells were exposed concomitantly to CPZ and light, cell viability dropped drastically to less than 30% at a concentration of approximately 2 µg/mL, whereas under dark conditions cell viability remained close to 100% (Figure 1A). In contrast, cells treated with SLS and light showed a similar cytotoxic behaviour as cells maintained under dark conditions (Figure 1B).

Subsequently, based on absorbance data from the NRU and MTT assays, IC_50_, PIF, and MPE values were determined using the OECD Phototox software (Table 3).

These results indicate that CPZ is classified as a phototoxic chemical according to both PIF (>5) and MPE values (>0.15), while SLS is classified as a non-phototoxic chemical, as expected.

### 3.2. Phototoxic Profile of the Selected Chemicals

**Figure 2:**
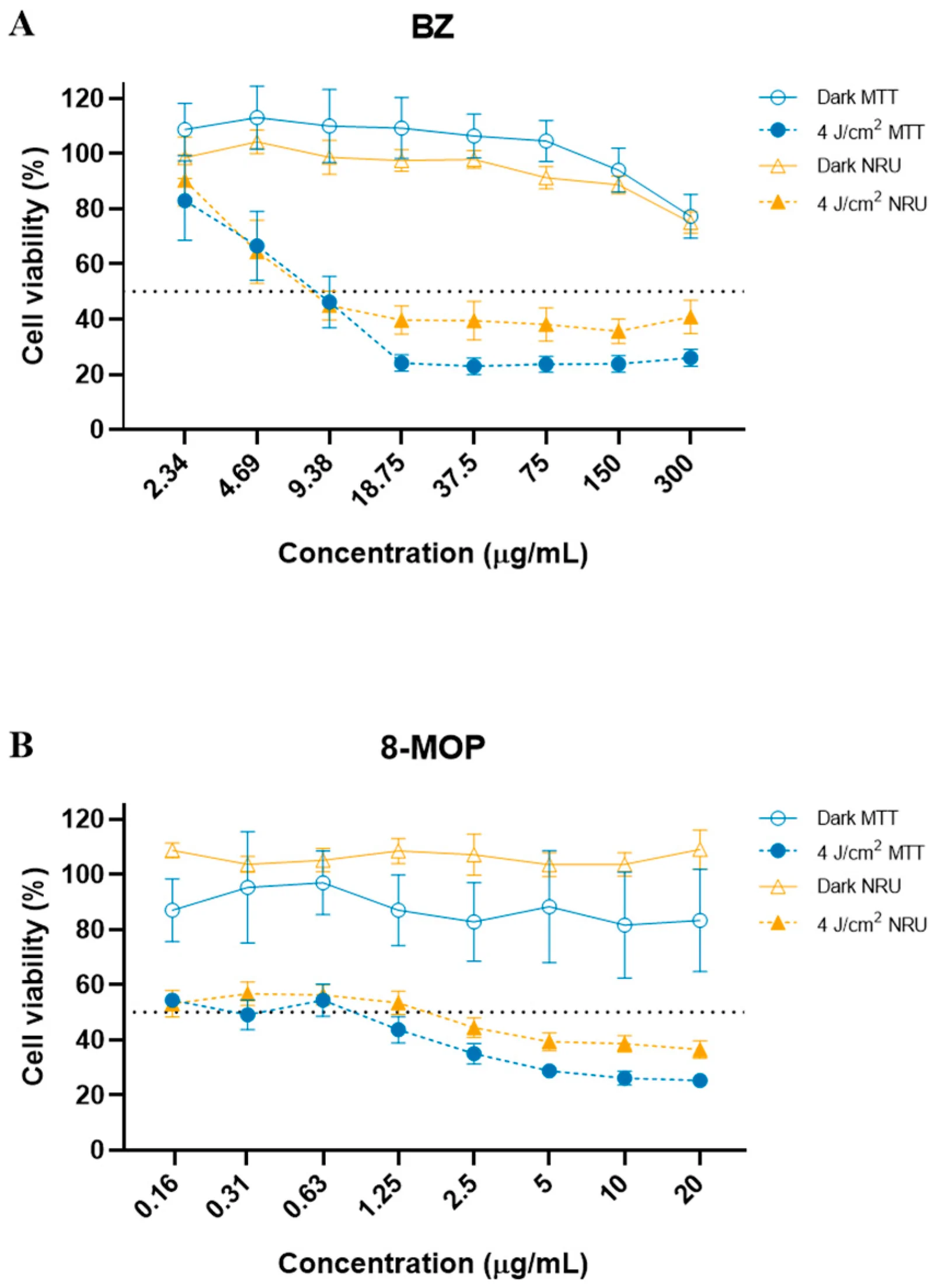

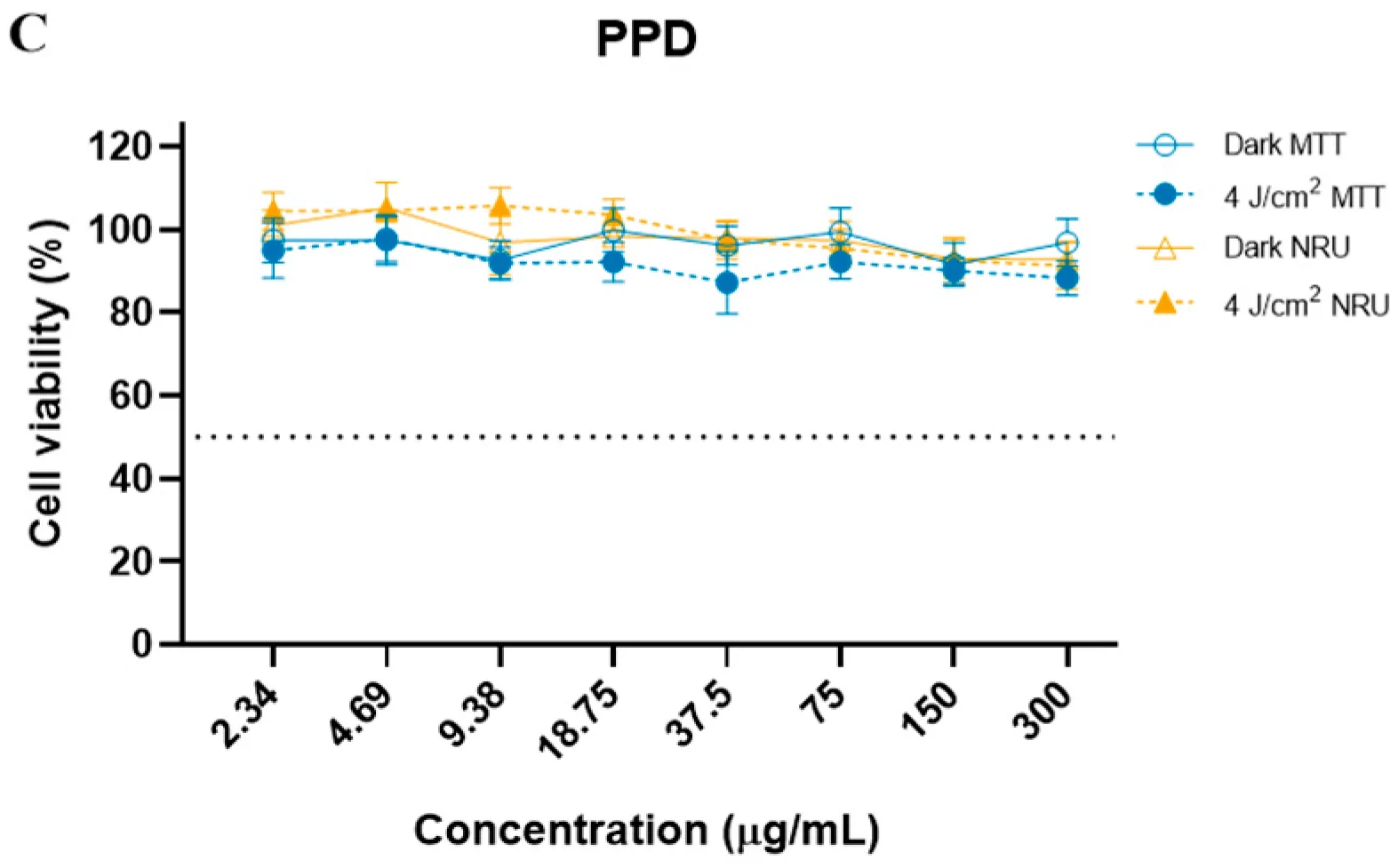
Cell viability of Benzophenone (BZ, (**A**)), 8-Methoxypsoralen (8-MOP, (**B**)) and *p*-phenylenediamine (PPD, (**C**)) in dark and irradiated conditions (4 J/cm^2^ of UVA light) obtained by the NRU and MTT tests. Results are expressed as mean ± SEM of 4–6-independent biological experiments.

The phototoxic activity of BZ, 8-MOP and PPD is presented in Figure 2, where cell viability was affected. In the presence of light, when cells were treated at non-cytotoxic or subtoxic concentrations of BZ and 8-MOP (Figure 2 A,B), this behaviour was not observed for PPD (Figure 2C). Nevertheless, data obtained for PPD should be regarded cautiously because at concentrations greater than 60 µg/mL oxidation of this chemical can interfere with spectrophotometric readings (see Figure S1 of the Supplementary Materials) and cell viability can be overestimated for this reason; the maximal concentration chosen for the next steps was fixed at 60 µg/mL.

When IC_50_, PIF, and MPE values were determined for these three chemicals (Table 4), their phototoxic potential was corroborated by both parameters, MPE and PIF.

**Figure 3:**
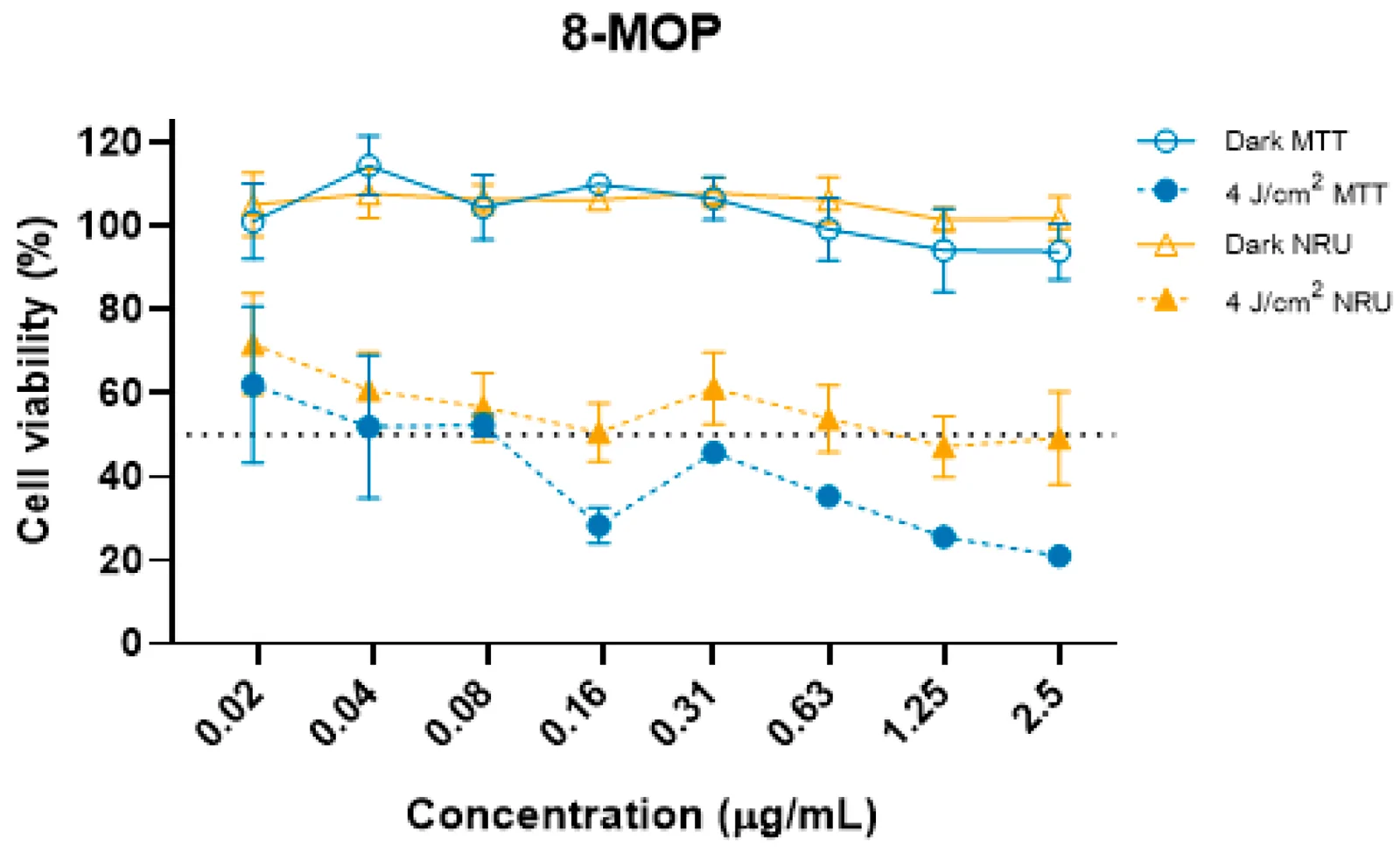
Cell viability of 8-Methoxypsoralen (8-MOP) at lower concentrations in dark and irradiated conditions (4 J/cm^2^ of UVA light) obtained by the NRU and MTT tests. Results are expressed as mean ± SEM of 4 independent biological experiments.

To better estimate the concentrations of 8-MOP that induce a cell viability ≥75–80%, further assays at lower concentrations were performed as shown in Figure 3. However, at the lowest concentration assessed (0.02 µg/mL), mean cell viabilities obtained were 72% ± 12.1 for the NRU and 62% ± 18.8 for the MTT assay. 8-MOP is highly insoluble and it was difficult to obtain a precise concentration with acceptable cell viability, but for the following studies, the maximal concentration used was determined to be 0.02 µg/mL.

### 3.3. IL-18 Production

Intracellular IL-18 levels determined by ELISA were normalized by the cellular protein content calculated by the Bradford assay as explained in Section 2.6. Moreover, to better understand how the different treatments and conditions affected the IL-18 production, a total stimulation index (TSI) was calculated by the equations below:

IL-18t non-irradiated/IL18c non-irradiated = NI(2)

IL-18t irradiated/IL18c irradiated = I(3)

TSI = I/NI(4)

where IL18 is the amount of IL-18 expressed in pg/mg protein obtained for each chemical treatment (t) or control cells (c); NI and I are the stimulation index of L-18 production in non-irradiated and irradiated conditions, respectively, for a specific chemical concentration, and TSI is the total stimulation index for such chemical concentration (see Table S1 in Supplementary Materials).

**Figure 4:**
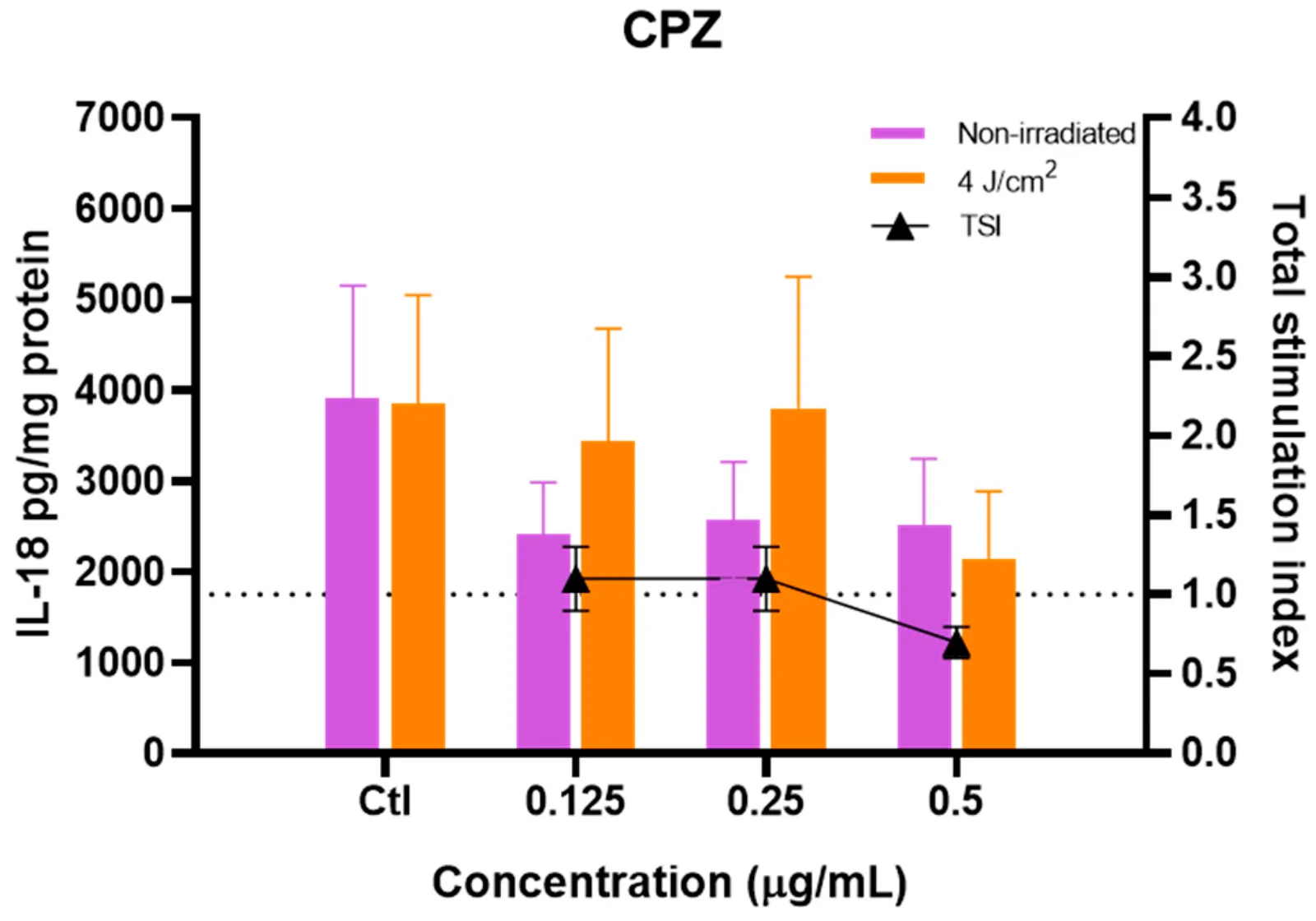
Intracellular IL-18 production by HaCaT treated with CPZ in non-irradiated and irradiated (4 J/cm^2^ of UVA) conditions expressed as pg/mg protein. Results are expressed as mean ± SEM of 4 independent biological experiments. Effect of different concentrations was determined using a two-way ANOVA followed by Dunnett’s post-hoc analysis.

The effect of CPZ on IL-18 production is presented in Figure 4, where a non-significant reduction can be observed at 0.125 µg/mL in dark conditions and at 0.5 µg/mL when cells are irradiated. The high variability of the amount of IL-18 determined in the different assays, as shown in Figure S2 for control cells, may explain why the decrease in IL-18 did not show statistically significant differences. Nevertheless, when TSI is calculated, a slight decrease was observed at 0.5 µg/mL (0.7 ± 0.1), suggesting a modest effect in the presence of UVA irradiation at such concentration.

**Figure 5:**
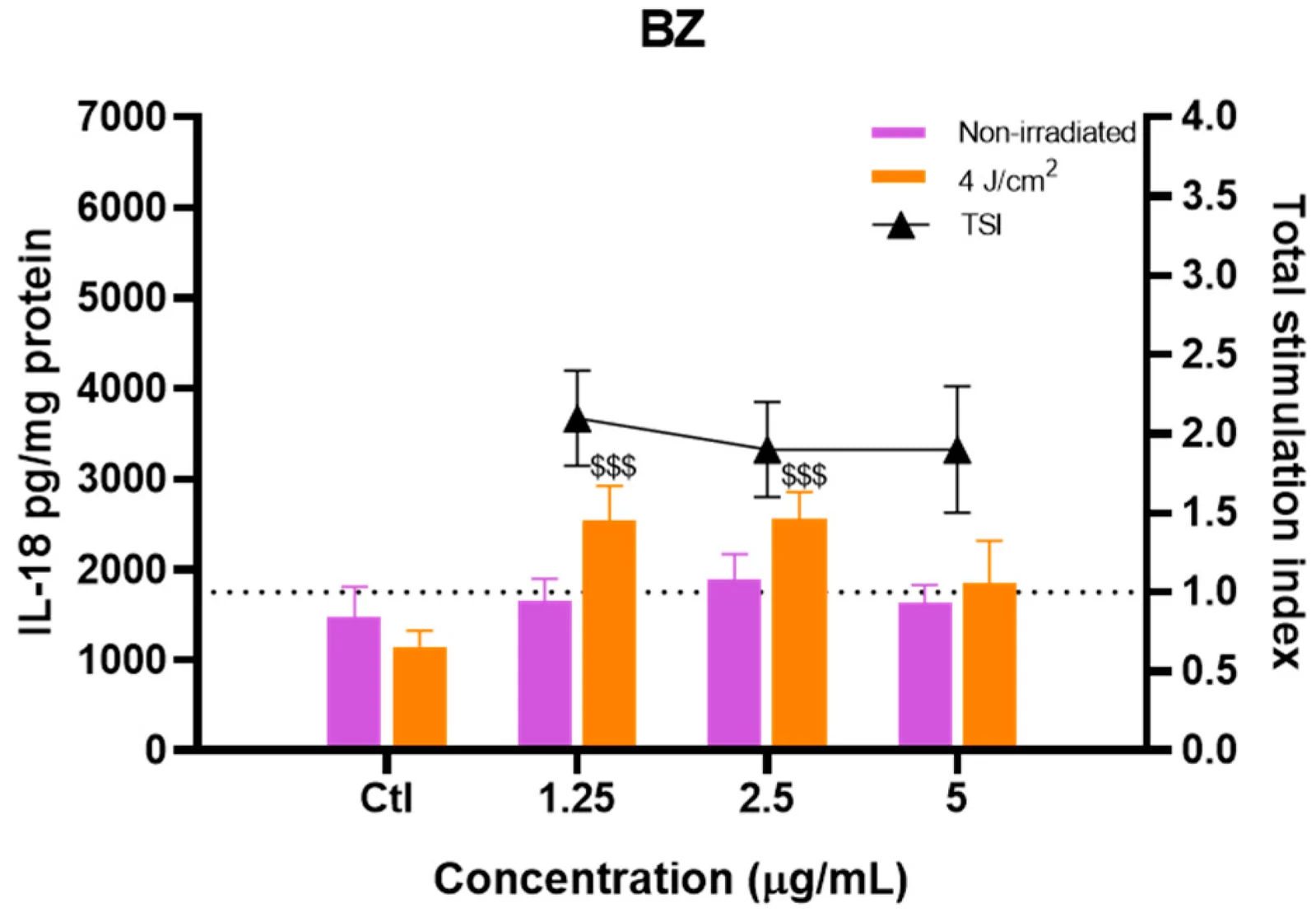
Intracellular IL-18 production by HaCaT treated with BZ in non-irradiated and irradiated (4 J/cm^2^ of UVA) conditions expressed as pg/mg protein. Results are expressed as mean ± SEM of 4 independent biological experiments. Effect of different concentrations was determined using a two-way ANOVA followed by Dunnett’s post-hoc analysis. Statistical differences between each concentration and its corresponding control under the same irradiation condition are indicated by ^$$$^ at *p* < 0.005.

In contrast, the exposure of HaCaT cells to BZ and UVA irradiation resulted in a significant increase in IL-18 production compared with irradiated control cells at 1.25 and 2.5 µg/mL (Figure 5, *p* < 0.005), whereas no effect of BZ was observed in cells maintained under dark conditions. Thus, statistical analyses indicate that there was a concomitant effect of BZ and light that was demonstrated by the value obtained for TSI, which is between 1.9 and 2.1 at the three concentrations considered.

**Figure 6:**
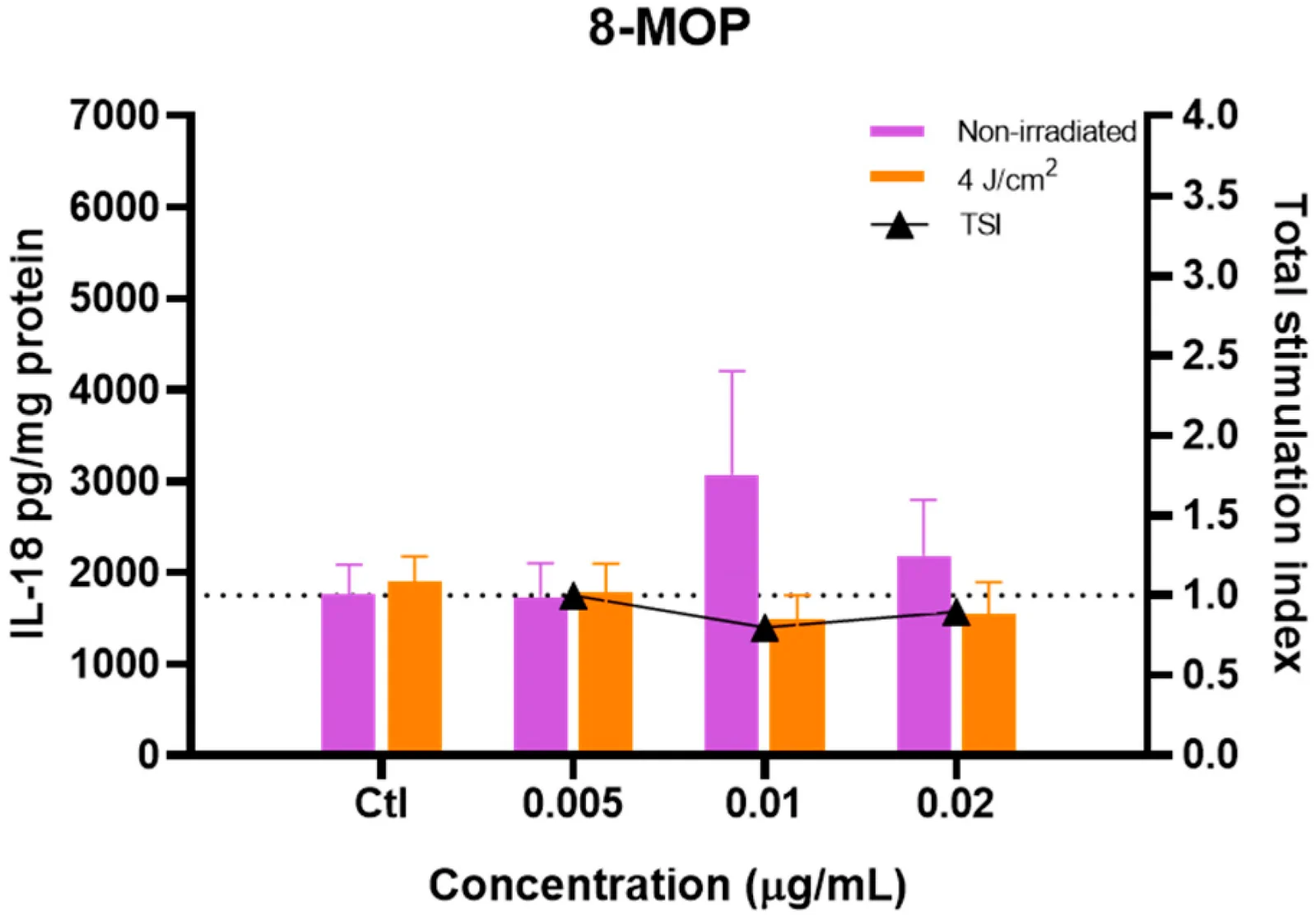
Intracellular IL-18 production by HaCaT treated with 8-MOP in non-irradiated and irradiated (4 J/cm^2^ of UVA) conditions expressed as pg/mg protein. Results are expressed as mean ± SEM of 4 independent biological experiments. Effect of different concentrations was determined using a two-way ANOVA followed by Dunnett’s post-hoc analysis.

Figure 6 shows the effect of 8-MOP on the production of IL-18. In this case, although a rise in IL-18 production was observed at 0.01 µg/mL in dark conditions, no statistically significant differences were observed in the conditions studied, as also reflected by the TSI calculated.

**Figure 7:**
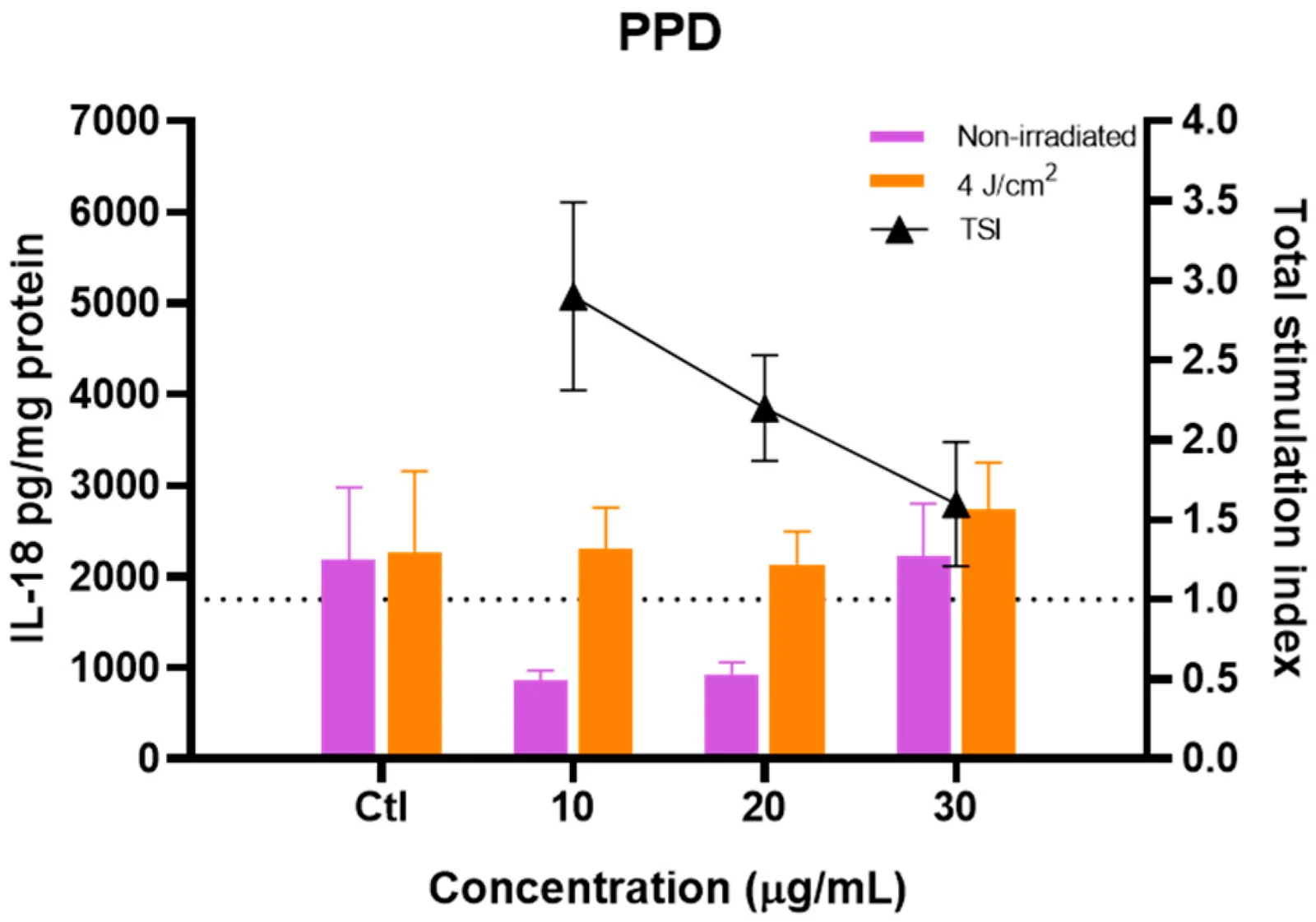
Intracellular IL-18 production by HaCaT treated with PPD in non-irradiated and irradiated (4 J/cm^2^ of UVA) conditions expressed as pg/mg protein. Results are expressed as mean ± SEM of 4 independent biological experiments. Effect of different concentrations was determined using a two-way ANOVA followed by Dunnett’s *post-hoc* analysis.

Finally, the effect of the allergen PPD is shown in Figure 7. This chemical appeared to induce the production of IL-18 when compared with the non-irradiated situation at concentrations of 10 and 20 µg/mL, which was also reflected by the high TSI values (2.9 and 2.2). However, this could be attributed to the not statistically significant lower production of IL-18 in dark conditions and not to a real induction of this interleukin in irradiation conditions because no statistically significant differences were found.

IL-18 levels were also quantified in the culture supernatants to evaluate whether secreted IL-18 could serve as a discriminatory biomarker. However, no significant differences were observed between treatments, indicating that extracellular IL-18 was not sufficiently sensitive under the experimental conditions used. In contrast, intracellular IL-18 displayed clear treatment-dependent modulation and was, therefore, selected as the primary endpoint for subsequent analyses.

### 3.4. Detection of Secreted Inflammatory Cytokines and MMPs

**Figure 8:**
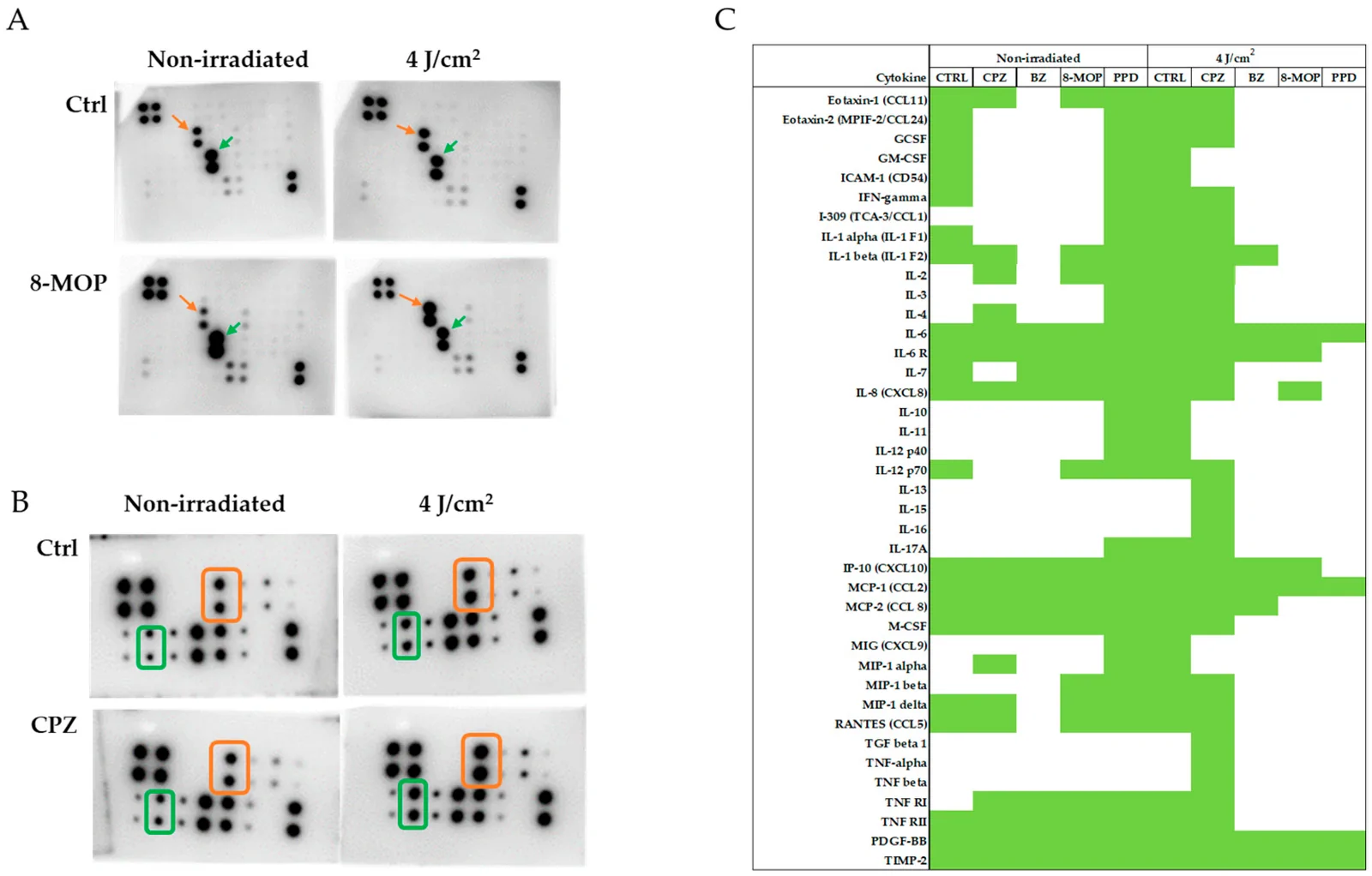
Exploratory identification of potential inflammatory cytokines and metalloproteinases as biomarkers of photoallergy and/or photoirritation. (**A**) Membrane arrays obtained after incubation of supernatants from control and 0.02 µg/mL 8-MOP treated cells in non-irradiated and irradiated conditions. Orange arrow identifies the signal for IL-6 and green for MCP-1. (**B**) Membrane arrays obtained after incubation of supernatants from control and 0.5 µg/mL CPZ-treated cells in non-irradiated and irradiated conditions. Orange square identifies the signal for MMP-1 and green for MMP-10. (**C**) Map of the chemiluminescence obtained for the two inflammatory membrane arrays where chemiluminescence signal is indicated in green.

An exploratory membrane-based antibody array was performed to identify candidate inflammatory mediators and/or metalloproteinases potentially modulated by the experimental treatment. As this assay was performed in a single experiment for screening purposes, the data were considered semi-quantitative and were not subjected to statistical analysis. In Figure 8, an example of membrane arrays for cytokine (A) and MMPs (B) detection is shown. In the case of inflammatory cytokines, and considering the two arrays performed, chemiluminescence signals were not detected for all 40 inflammatory cytokines, as shown in Figure 8C. As observed, in the case of non-irradiated control cells, only 55% of the cytokines were detected. This percentage was slightly reduced in the presence of the chemicals (detection of 30–48% of cytokines) except in the case of PPD (85%). Light irradiation per se increased the number of detected inflammatory cytokines (85%), but chemicals reduced the signal to 10–20%, except in the case of CPZ (83%). Consequently, only signals for IL-6, MCP-1, TNF RII, PDGF-BB and TIMP-2 were detected in all the conditions assayed. These preliminary results suggest that potential biomarkers related to photoirritation and photoallergenicity are IL-6, MCP-1, TNF RII, PDGF-BB and TIMP-2. The normalized chemiluminescence is shown in Figures S3 and S4 of the Supplementary Materials. In the case of MMPs, chemiluminescence signals were detected in all conditions studied.

**Figure 9:**
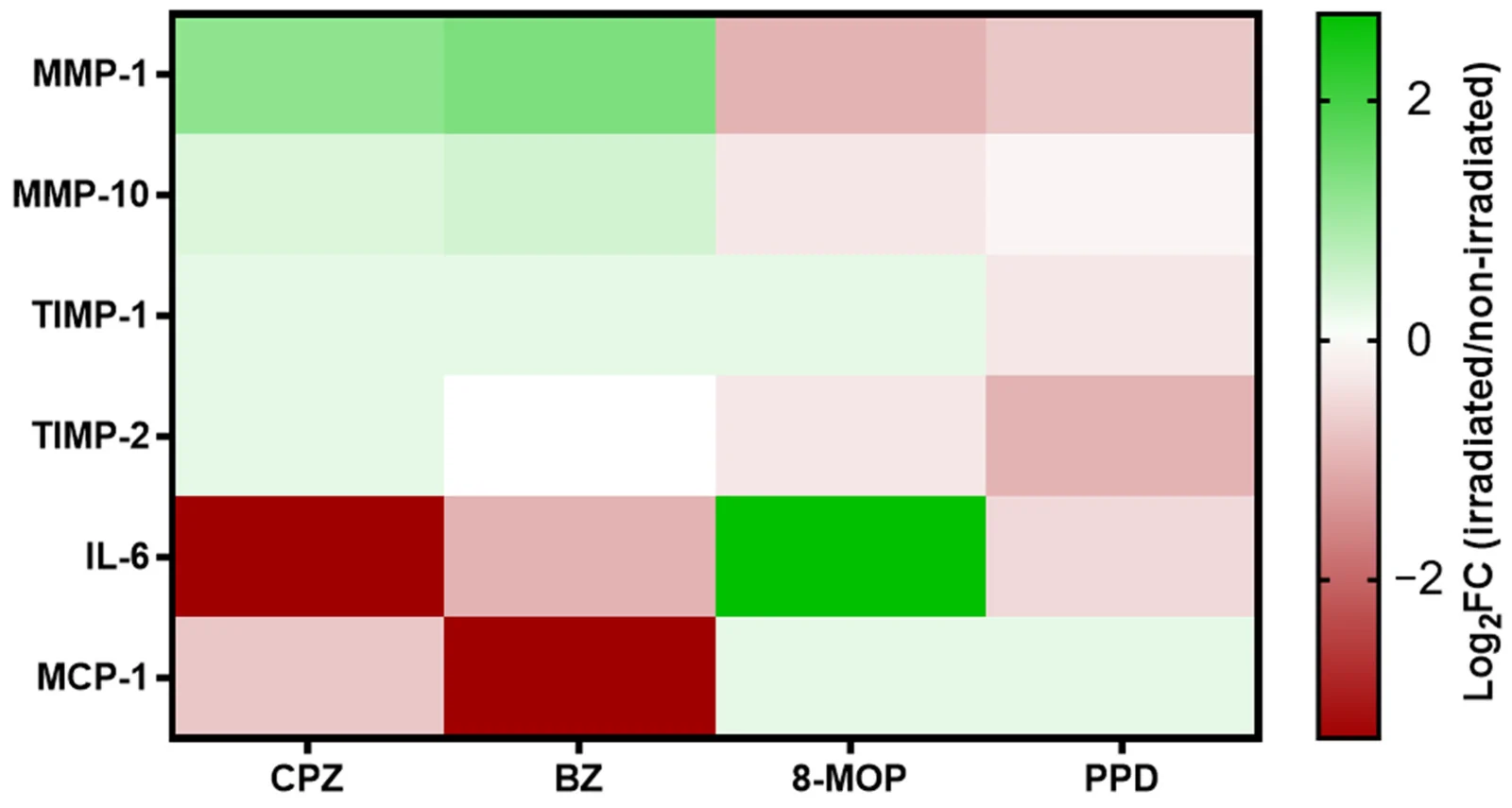
Heat map showing the log_2_ fold changes (log_2_FC) of interleukins and MMPs signal intensities in irradiated samples relative to non-irradiated controls. Log_2_FC values were calculated as log_2_(irradiated/non-irradiated intensity). Negative values indicate decreased signal intensity after irradiation, whereas positive values indicate increased signal intensity. Values around zero represent no changes between irradiated and non-irradiated conditions. The color scale represents the magnitude and direction of these changes, with red indicating negative values, white indicating values close to zero, and green indicating positive values.

To facilitate data interpretation, Figure 9 presents a heat map of the inflammatory cytokines and MMPs displaying the strongest chemiluminescent signals and the most pronounced irradiation-induced changes. Analytes with signal intensities close to background levels were excluded, as large fold changes derived from low-intensity signals may not reflect biologically relevant effects.

As shown in Figure 9, CPZ and BZ upregulated the production of MMP-1, MMP-10, TIMP-1 and TIMP-2 while downregulating the release of IL-6 and MCP-1, whereas 8-MOP exhibited the opposite pattern. Nevertheless, cell viability at the selected concentration of 8-MOP fell below the predefined 75–80% threshold and should, therefore, be considered when interpreting the corresponding biomarker responses. Finally, PPD decreased the release of almost all soluble mediators shown except for a discrete increase in MCP-1. From these data, we finally considered that the most promising candidates for further studies should be MMP-1, MMP-10 and IL-6. However, considering the detected signal (see Figures S5 and S6), MMP-1 appeared to be more promising than MMP-10.

## 4. Discussion

Driven by the ban on animal testing for cosmetics and by growing scientific and regulatory demands, the development of alternative methods has become a critical priority for assessing chemical toxicity. Moreover, the use of human-relevant assays is crucial for improving our understanding of the mechanisms of action underlying toxic responses. In this context, the identification and characterization of key events within adverse outcome pathways (AOPs) have emerged as a central objective in modern toxicology. This strategy aligns closely with the global shift towards new approach methodologies (NAMs), which demands the design and development of novel high-throughput tools capable of predicting potential hazards independently of animal models [23].

Phototoxicity represents an important toxicological concern worldwide, particularly for substances intended for topical application and exposure to sunlight. Currently, established OECD guidelines, such as the Organisation for Economic Co-operation and Development Test Guideline 432 for the in vitro 3T3 NRU phototoxicity assay and the ROS assay guideline, provide validated approaches for predicting the phototoxic potential of chemicals [8,12]. However, despite the usefulness of these methods, neither can distinguish phototoxic compounds (photoirritants) from photoallergens. Such discrimination is essential for improving hazard assessment and ensuring safer development of cosmetic and pharmaceutical ingredients.

To evaluate the predictive capacity of the adapted OECD TG 432 protocol using the HaCaT cell line, the phototoxicity of well-characterized reference compounds was assessed by calculating both the PIF and MPE values. Cell viability was measured using both the guideline-recommended NRU assay [12] and the MTT assay. The results obtained with CPZ as well as with SLS, both reference compounds recommended in OECD TG 432, confirmed its classification as phototoxic and non-phototoxic. This classification was achieved not only with the NRU assay, yielding PIF and MPE values within the ranges specified in the guideline, but also with the MTT assay. Furthermore, the additional chemicals assessed (BZ, 8-MOP and PPD) were correctly classified using the adapted HaCaT-based method.

These findings suggest that replacing the Balb/c 3T3 cell line with HaCaT keratinocytes does not compromise the ability of the assay to identify phototoxic substances [24]. Moreover, the key role of keratinocytes in the early stages of the cutaneous response to photoallergens [5] could represent a valuable first step towards identifying candidate biomarkers associated with the onset of photocontact allergy.

Keratinocytes play a central role in the initiation and regulation of cutaneous immune responses, including the production of interleukin-18 (IL-18), a key cytokine involved in the induction of allergic contact sensitization [25]. Previous studies have demonstrated IL-18 production in human keratinocytes, including the NCTC 2544 cell line [26,27], as well as in reconstructed human epidermis models [28,29] when treated with contact allergens. These findings support the use of IL-18 as a promising biomarker for the identification of contact allergens, allowing their discrimination from respiratory allergens and irritants. Moreover, increased IL-18 production has also been reported following exposure of keratinocytes to well-known photoallergens, suggesting that this cytokine may be involved in photoallergic responses and could represent a promising biomarker for the identification of photoallergic potential [14,30], suggesting a potential role for this cytokine in photoallergic responses.

In the present study, we further investigate the modulation of IL-18 production in keratinocytes exposed to photoallergens, using the HaCaT cell line, a well-characterized immortalized human keratinocyte model widely employed in dermatological and toxicological research [31,32,33,34,35]. Although CPZ, BZ and 8-MOP are recognized phototoxic compounds, discrepancies remain regarding their specific phototoxic profiles and the relative contribution of photoirritant and photoallergic mechanisms, highlighting the complexity of classifying phototoxic compounds according to their predominant mode of action. The identification of reliable biomarkers capable of discriminating between these two mechanisms would, therefore, represent a valuable tool for improving characterization of phototoxic responses and the identification of photoallergic potential. The lack of discriminatory changes in secreted IL-18, together with the more pronounced intracellular modulation observed in our model, suggests that intracellular IL-18 may represent a more sensitive endpoint for identifying photoallergic responses under the experimental conditions employed. This finding supports the use of intracellular IL-18 as the primary biomarker in the proposed assay, although further studies are warranted to determine whether secreted IL-18 may be informative under different experimental settings or exposure conditions.

Several tested compounds illustrate this challenge. The capacity of benzophenone to induce photocontact sensitization is well established [36,37] and it is accordingly classified as a photoallergen in several studies [16,17,38]. However, other reports have suggested that benzophenone may induce both photoallergic and photoirritant responses [39,40], indicating that its phototoxic profile may depend on the experimental conditions and the biological model employed. Notably, benzophenone was the only compound in our study to show a clear increase in IL-18 production following UVA irradiation, a response consistent with its reported photoallergic potential and one that further supports the involvement of IL-18 in detecting photoallergic mechanisms.

Similarly, 8-methoxypsoralen has been mainly described as a photoirritant compound [16,18,19], although some studies have also suggested a potential photoallergic activity [39,40], indicating a potential dual phototoxic behaviour. In agreement with our findings, 8-MOP exhibited a predominantly photoirritant profile under the experimental conditions evaluated, as no relevant increase in IL-18 production was observed following UVA irradiation. Alternatively, if 8-MOP possesses some degree of photoallergic potential [21], its photoirritant activity appears to prevail over its photoallergic response in this model.

Chlorpromazine showed an intermediate behaviour, consistent with its classification as both a photoirritant and photoallergen compound [16,17,18,22,38,39]. Unexpectedly, CPZ reduced intracellular IL-18 levels under dark conditions. However, the biological significance of this finding remains unclear, and the available literature does not provide sufficient evidence to explain this response in the absence of UVA irradiation. Therefore, no mechanistic conclusions can be drawn from the present study, and this observation should be interpreted with caution pending further mechanistic investigation.

Finally, PPD is widely recognized as a contact allergen [16,41] that has been proposed as a reference chemical to demonstrate the viability of identifying contact sensitizers in vitro [27]. Although occasional reports have described photosensitivity or photoaggravation associated with PPD exposure, it is not generally regarded as a reference photoallergen. Therefore, PPD was intentionally included as a representative contact allergen to distinguish contact allergenicity from photoallergic responses in our model [42,43]. In our case, the concomitant exposure of keratinocytes to PPD and UVA did not induce any real increase in the intracellular content of IL-18 as previously reported [14,30].

Studies involving NCTC2544 keratinocytes [14] demonstrated that determination of intracellular IL-18 can be a useful tool for distinguishing between photoirritants (8-MOP) and photoallergens (BZ). However, that model identified CPZ as a photoallergen rather than a photoirritant, unlike our study. Differences in the protocols used, such as contact time between the chemicals as well as the different cell profiles, could explain these slight discrepancies [44,45].

Overall, this exploratory proof-of-concept study suggests that intracellular IL-18 may represent a promising biomarker contributing to the identification of photoallergic responses in keratinocytes. However, the heterogeneous behaviour observed among the tested compounds, together with discrepancies reported in previous studies, indicates that IL-18 alone may not be sufficient to unequivocally discriminate photoallergens from photoirritants in all cases, as keratinocyte monocultures cannot fully reproduce the complex immune mechanisms involved in photoallergic reactions. Rather, IL-18 may constitute one component of a broader biomarker panel that requires further validation using a larger set of reference compounds and more physiologically relevant experimental models.

The discrepancies observed between our findings and previous reports also highlight the influence of experimental design on IL-18 responses. Factors such as the keratinocyte model employed (HaCaT versus NCTC2544), exposure conditions, irradiation protocol, UVA dose, incubation time and endpoint selection (intracellular versus secreted IL-18) may all contribute to differences in biomarker performance. Consequently, caution should be exercised when comparing studies directly, and further harmonization of experimental protocols will be essential to establish the robustness of IL-18 as a biomarker of photoallergic potential.

A relatively high inter-experimental variability in basal intracellular IL-18 levels among control cells was observed throughout the study, as shown in Figure S2. Similar variability has previously been reported [14,27], as factors such as cell density, confluence, passage number, and other intrinsic cellular characteristics may influence IL-18 production. To minimize these sources of variability, all experiments were performed using the same seeding density, and cells were monitored microscopically to ensure comparable confluence before treatment. Accordingly, maintaining low and comparable passage numbers should also be considered an important factor for improving the reproducibility of this assay. Importantly, within each independent experiment, basal IL-18 levels were comparable between non-irradiated and irradiated control cells, indicating that UVA exposure alone did not substantially affect intracellular IL-18 production. Therefore, treatment effects were evaluated relative to the corresponding non-irradiated and irradiated controls within each independent experiment rather than by comparing absolute IL-18 values across experiments, thereby minimizing the impact of inter-experimental variability on data interpretation.

To explore novel biomarkers for the identification of photoallergens and photoirritants, exploratory membrane-based antibody arrays were performed. Interestingly, an increase in the secretion of MMP-1 and MMP-10 (in a modest manner) was observed following UVA exposure in cells treated with chlorpromazine and benzophenone. In contrast, Interleukin-6 secretion was increased in cells treated with 8-methoxypsolaren after UVA exposure, whereas supernatants with chlorpromazine and benzophenone showed a marked downregulation of this cytokine. This differential cytokine pattern indicates that compounds with photoirritant potential may upregulate the secretion of the pro-inflammatory cytokine interleukin-6, whereas compounds with photoallergic (or borderline) potential may preferentially induce MMP-1 expression.

Within the proposed AOP for photoallergic sensitization by de Ávila et al. [6], protein reactivity leading to hapten-protein complex formation represents the first key event, followed by keratinocyte activation and the subsequent activation of downstream inflammatory pathways. Therefore, the biomarkers evaluated in the present study (IL-18, IL-6 and MMP-1) should be regarded as indicators of keratinocyte activation and downstream cellular responses rather than direct measures of the protein-reactivity potential of photoactivated chemicals. Accordingly, integrating assays that assess protein reactivity with downstream cellular biomarkers may provide a more comprehensive mechanistic evaluation of photoallergic potential by linking early and late key events within the AOP.

UVA exposure induces the production of intracellular ROS that stimulates the secretion of inflammatory mediators and increases MMP activity, among other processes [46]. Also, in contact allergy, intracellular ROS production has been shown to promote the release of damage-associated molecular patterns (DAMPs), such as HMGB1, which can trigger hyaluronan degradation. The resulting hyaluronan fragments have been implicated in the induction of IL-18 production [47]. Therefore, a similar mechanism may underlie the increase in IL-18 observed after photoallergen exposure, whereby UVA-induced ROS promote hyaluronan degradation and subsequently enhance IL-18 expression. Moreover, we proposed other mediators that can be upregulated distinctly by photoallergens or photoirritants. Thus, IL-6 secretion in the presence of photoirritants and UVA light can be explained by its role in the skin inflammatory process after UVA [48] and irritant exposure [49,50]. While MMP-1 induction during photoaging is well established [51,52], the marked increase observed in the present study cannot be fully explained by this mechanism alone. Rather, it more plausibly reflects a synergistic interaction between the photoallergen and UVA irradiation. This interpretation is further supported by previous reports implicating MMP-1 as a contributing factor in allergic skin conditions [53], reinforcing the notion that this MMP plays an active role in the photoallergic response.

Although IL-6 has been proposed as a mediator of allergic contact dermatitis and has been reported to increase in keratinocytes exposed to contact allergens [50,54,55], the present findings indicate a different pattern under photoallergic conditions, where UVA irradiation may modulate cytokine responses. Moreover, these observations are based on a limited set of reference chemicals and should, therefore, be considered preliminary. Further studies including a broader panel of photoallergens and photoirritants are in progress to determine whether IL-6 has value as a discriminatory biomarker in photosafety assessment.

Accordingly, MMP-1 and IL-6 emerged as preliminary candidate biomarkers that may complement intracellular IL-18 in distinguishing different phototoxic response patterns under the experimental conditions evaluated. However, these findings are based on a limited number of reference compounds and should be regarded as exploratory proof-of-concept observations. Additional studies including a larger panel of well-characterized photoallergens and photoirritants will be required to independently validate their discriminatory value and assess their applicability in predictive testing strategies. Future studies will also explore alternative cell viability assays, such as PrestoBlue or Alamar Blue, which may facilitate subsequent quantification of intracellular and extracellular interleukins by ELISA from the same experimental samples.

## 5. Conclusions

The present study demonstrates that the proposed model can discriminate between phototoxic and non-phototoxic chemicals. Beyond its predictive capacity, the model offers several potential advantages, including improved human relevance and the possibility of evaluating mechanistically relevant biomarkers that may enable the discrimination between photoirritant and photoallergic responses when integrated with other assays.

Moreover, the assay is amenable to high-throughput screening, enabling the efficient evaluation of large chemical libraries. This is consistent with current European regulatory initiatives actively promoting the development and implementation of New Approach Methodologies (NAMs) within integrated testing and assessment strategies [56].

Despite these promising findings, further studies are required before the model can be considered for regulatory implementation. Ongoing work is focused on evaluating a larger and more diverse panel of chemicals covering a broad range of phototoxicity profiles, while simultaneously assessing the proposed novel biomarkers (IL-6 and MMP-1) using ELISA-based approaches. The outcomes of these studies will be essential to establish the robustness, reproducibility, and added value of the model, ultimately consolidating its potential as a reliable tool within integrated testing strategies.

Should these additional studies confirm its predictive performance and mechanistic relevance, the model could be incorporated into a tiered Next Generation Risk Assessment (NGRA) strategy if validated. Within such a framework, an initial UV/Vis assessment could be followed by testing with the proposed model, with positive or equivocal results subsequently confirmed using a reconstructed human epidermis (RhE)-based assay [57]. This integrated tiered approach holds strong potential to enhance both the human relevance and efficiency of photosafety assessment, while actively supporting the transition towards non-animal testing strategies.

## Figures and Tables

**Table 2:** Percentage of cell viability after exposure to UVA light obtained with the NRU and MTT assays.

UVA Dose	NRU ^1^	MTT ^1^
5 J/cm^2^	85.7 ± 3.3	66.9 ± 7.7
4 J/cm^2^	86.1 ± 1.6	84.7 ± 1.6

^1^ Results are expressed as mean ± SEM of 6 technical replicates.

**Table 3:** IC_50_, PIF and MPE * obtained for CPZ and SLS as proficiency chemicals.

Chemical	Assay	IC_50_		PIF	MPE
		Non-Irradiated	Irradiated		
CPZ	NRU	n.d.	0.9 ± 0.3 µg/mL	>42.1 ± 12.3	0.49 ± 0.14
	MTT	n.d.	1.1 ± 0.1 µg/mL	>29.1 ± 2.8	0.40 ± 0.06
SLS	NRU	27.0 ± 3.9 µg/mL	27.2 ± 2.0 µg/mL	1.0 ± 0.1	−0.04 ± 0.05
	MTT	25.2 ± 2.2 µg/mL	24.1 ± 2.8 µg/mL	1.1 ± 0.1	−0.01 ± 0.03

* Results are expressed as mean ± SEM of 4–5 independent biological experiments. n.d. denotes that IC_50_ could not be calculated as minimum cell viability at maximal concentration assayed is > 50%.

**Table 4:** IC_50_, PIF and MPE * obtained for BZ, 8-MOP and PPD.

Chemical	Assay	IC_50_		PIF	MPE
		Non-Irradiated	Irradiated		
BZ	NRU	n.d.	5.6 ± 1.3 µg/mL	>62.5 ± 14.3	0.36 ± 0.07
	MTT	n.d.	8.7 ± 1.1 µg/mL	>37.9 ± 6.5	0.56 ± 0.09
8-MOP	NRU	n.d.	0.3 ± 0.1 µg/mL	>372.1 ± 243.5	0.68 ± 0.03
	MTT	n.d.	0.3 ± 0.07 µg/mL	>169.21 ± 75.4	0.66 ± 0.01
PPD	NRU	n.d.	n.d.	1.0 ± 0.0	0.02 ± 0.10
	MTT	n.d.	n.d.	1.0 ± 0.0	0.10 ± 0.09

* Results are expressed as mean ± SEM of 4–6 independent biological experiments. n.d. denotes that IC_50_ could not be calculated as minimum cell viability at maximal concentration assayed is >50%.

## Data Availability

The data supporting the findings of this study are available within this article and its Supplementary Materials. The raw data from individual experiments and replicates are available from the corresponding author upon reasonable request.

## Supplementary Materials

The following supporting information can be downloaded at: https://www.mdpi.com/article/10.3390/toxics14080726/s1, Figure S1: Interferences of PPD with MTT readings; Figure S2: Intracellular IL-18 production by non-irradiated and irradiated at 4 J/cm^2^ control cells across the experiments; Figure S3: Chemiluminescence signals obtained with the cytokine inflammatory membrane arrays; Figure S4: Relationship of inflammatory cytokine chemiluminescence signals obtained after each treatment with the respective control; Figure S5: Chemiluminescence signals obtained with the metalloproteinase inflammatory membrane arrays; Figure S6: Relationship of metalloproteinase chemiluminescence signals obtained after each treatment with the respective control; Table S1: Stimulation indexes in non-irradiated and irradiated conditions for IL-18.

## Abbreviations

The following abbreviations are used in this manuscript:

8-MOP	8-methoxypsoralen
AOP	Adverse outcome pathway
BSA	Bovine serum albumin
BZ	Benzophenone
CPZ	Chlorpromazine hydrochloride
DAMPs	Damage-associated molecular patterns
DMEM	Dulbecco’s Modified Eagle’s Medium
DMSO	Dimethyl sulfoxide
DTH	Delayed-type hypersensitivity
ECL	Enhanced chemiluminescence
ELISA	Enzyme Linked Immunosorbent Assay
FBS	Fetal bovine serum
HRP	Horseradish peroxidase
I	Stimulation index in irradiated conditions
IC_50_	concentration that induces 50% of mortality
IL-18	Interleukin 18
ITS	Integrated test strategies
MEC	Molar Extinction Coefficient
MMP-1	Metalloproteinase-1
MMPs	Matrix metalloproteinases
MPE	Mean Photo Effect
MTT	3-[4,5-dimethylthiazol-2-yl]-2,5 diphenyl tetrazolium bromide
NAMs	New approach methodologies
NI	Stimulation index in non-irradiated conditions
NRU	Neutral red uptake
OECD	Organisation for Economic Co-operation and Development
PBS	Phosphate buffer solution
PIF	Photo Irritation Factor
PPD	*p*-phenylendiamine
RhE	Reconstructed human epidermis
ROS	Reactive oxygen species
SLS	Sodium lauryl sulphate
TSI	Total stimulation index
UV	Ultraviolet
UV/Vis	Ultraviolet/Visible

## References

[1] Pai S.B., Anchan V.N. (2024). Phototoxicity and photoallergy—A brief review on pathogenesis, recent advances, and approach to a patient with photosensitivity. Indian J. Skin. Allergy.

[2] Hofmann G.A., Weber B. (2021). Drug-induced photosensitivity: Culprit drugs, potential mechanisms and clinical consequences. J. Dtsch. Dermatol. Ges..

[3] Onoue S., Seto Y., Sato H., Nishida H., Hirota M., Ashikaga T., Api A.M., Basketter D., Tokura Y. (2017). Chemical photoallergy: Photobiochemical mechanisms, classification, and risk assessments. J. Dermatol. Sci..

[4] Kutlubay Z., Sevim A., Engin B., Tüzün Y. (2014). Photodermatoses, including phototoxic and photoallergic reactions (internal and external). Clin. Dermatol..

[5] OECD (2014). The Adverse Outcome Pathway for Skin Sensitisation Initiated by Covalent Binding to Proteins.

[6] de Ávila R.I., Aleksic M., Zhu B., Li J., Pendlington R., Valadares M.C. (2023). Non-animal approaches for photoallergenicity safety assessment: Needs and perspectives for the toxicology for the 21st century. Regul. Toxicol. Pharmacol..

[7] OECD (1981). Test No. 101: UV-VIS Absorption Spectra. OECD Guidelines for the Testing of Chemicals, Section 1.

[8] OECD (2019). Test No. 495: Ros (Reactive Oxygen Species) Assay for Photoreactivity. OECD Guidelines for the Testing of Chemicals, Section 4.

[9] OECD (2026). Test No. 442C: In Chemico Skin Sensitisation: Assays addressing the Adverse Outcome Pathway key event on covalent binding to proteins. OECD Guidelines for the Testing of Chemicals, Section 4.

[10] Maddaleno A.S., Vinardell M.P., Mitjans M. (2024). Innovative Strategies for Photoallergy Assessment: Breaking Free from Animal Models in Cosmetic Ingredient Development. Cosmetics.

[11] Boukamp P., Petrussevska R.T., Breitkreutz D., Hornung J., Markham A., Fusenig N.E. (1988). Normal keratinization in a spontaneously immortalized aneuploid human keratinocyte cell line. J. Cell Biol..

[12] OECD (2019). Test No. 432: In Vitro 3T3 NRU Phototoxicity Test. OECD Guidelines for the Testing of Chemicals, Section 4.

[13] Martínez V., Galbiati V., Corsini E., Martín-Venegas R., Vinardell M.P., Mitjans M. (2013). Establishment of an in vitro photoassay using THP-1 cells and IL-8 to discriminate photoirritants from photoallergens. Toxicol. In Vitro.

[14] Galbiati V., Martínez V., Bianchi S., Mitjans M., Corsini E. (2013). Establishment of an in vitro photoallergy test using NCTC2544 cells and IL-18 production. Toxicol. In Vitro.

[15] Patel N.H., Mishra P.K., Nagane R., Deshpande A., Tamboli I.Y., Date R. (2019). Comparison of in chemico skin sensitization methods and development of an in chemico skin photosensitization assay. Altern. Anim. Exp..

[16] Nguyen R., Barry M., Azevedo Loiola R., Ferret P.J., Andres E. (2023). PhotoSENSIL-18 assay development: Enhancing the safety testing of cosmetic raw materials and finished products to support the in vitro photosensitization assessment?. Toxicology.

[17] Tsujita-Inoue K., Hirota M., Atobe T., Ashikaga T., Tokura Y., Kouzuki H. (2016). Development of novel in vitro photosafety assays focused on the Keap1-Nrf2-ARE pathway. J. Appl. Toxicol..

[18] Yamamoto Y., Fujita M., Wanibuchi S., Sato A., Katsuoka Y., Kasahara T. (2020). Development of photo-amino acid derivative reactivity assay: A novel in chemico alternative method for predicting photoallergy. J. Appl. Toxicol..

[19] Man I., Crombie I.K., Dawe R.S., Ferguson J., Ibbotson S.H. (2004). The optimal time to determine the minimal phototoxic dose in skin photosensitized by topical 8-methoxypsoralen. Br. J. Dermatol..

[20] Bonamonte D., Foti C., Lionetti N., Rigano L., Angelini G. (2010). Photoallergic contact dermatitis to 8-methoxypsoralen in Ficus carica. Contact Dermat..

[21] Plewig G., Hofmann C., Braun-Falco O. (1978). Photoallergic dermatitis from 8-methoxypsoralen. Arch. Dermatol. Res..

[22] Toyoda A., Itagaki H. (2018). Development of an in vitro photosafety evaluation method utilizing intracellular ROS production in THP-1 cells. J. Toxicol. Sci..

[23] Sewell F., Alexander-White C., Brescia S., Currie R.A., Roberts R., Roper C., Vickers C., Westmoreland C., Kimber I. (2024). New approach methodologies (NAMs): Identifying and overcoming hurdles to accelerated adoption. Toxicol. Res..

[24] Svobodová A.R., Zálešák B., Biedermann D., Ulrichová J., Vostálová J. (2016). Phototoxic potential of silymarin and its bioactive components. J. Photochem. Photobiol. B.

[25] Corsini E., Gibbs S., Roggen E., Kimber I., Basketter D.A., Palmeira C.M.M., de Oliveira D.P., Dorta D.J. (2021). Skin Sensitization Tests: The LLNA and the RhE IL-18 Potency Assay. Toxicity Assessment: Methods in Molecular Biology.

[26] Corsini E., Mitjans M., Galbiati V., Lucchi L., Galli C.L., Marinovich M. (2009). Use of IL-18 production in a human keratino-cyte cell line to discriminate contact sensitizers from irritants and low molecular weight respiratory allergens. Toxicol. In Vitro.

[27] Galbiati V., Mitjans M., Lucchi L., Viviani B., Galli C.L., Marinovich M., Corsini E. (2011). Further development of the NCTC 2544 IL-18 assay to identify in vitro contact allergens. Toxicol. In Vitro.

[28] Gibbs S., Corsini E., Spiekstra S.W., Galbiati V., Fuchs H.W., DeGeorge G., Troese M., Hayden P., Deng W., Roggen E. (2013). An epidermal equivalent assay for identification and ranking potency of contact sensitizers. Toxicol. Appl. Pharmacol..

[29] Galbiati V., Papale A., Marinovich M., Gibbs S., Roggen E., Corsini E. (2017). Development of an in vitro method to estimate the sensitization induction level of contact allergens. Toxicol. Lett..

[30] Galbiati V., Bianchi S., Martínez V., Mitjans M., Corsini E. (2014). NCTC 2544 and IL-18 production: A tool for the in vitro identification of photoallergens. Toxicol. In Vitro.

[31] Chung H., Quan H., Jung D., Ravi G., Cho A., Kang M.J., Kim E., Che J.H., Lee E.S., Jeong T.C. (2018). Intra- and inter-laboratory reproducibility and predictivity of the HaCaSens assay: A skin sensitization test using human keratinocytes, HaCaT. Toxicol. In Vitro.

[32] Eskes C., Hennen J., Schellenberger M.T., Hoffmann S., Frey S., Goldinger-Oggier D., Peter N., Van Vliet E., Blömeke B. (2019). The HaCaT/THP-1 Cocultured Activation Test (COCAT) for skin sensitization: A study of intra-lab reproducibility and predictivity. Altern. Anim. Exp..

[33] de Ávila R.I., Veloso D.F.M.C., Teixeira G.C., Rodrigues T.L., Lindberg T., Lindstedt M., Fonseca S.G., Lima E.M., Valadares M.C. (2019). Evaluation of in vitro testing strategies for hazard assessment of the skin sensitization potential of “real-life” mixtures: The case of henna-based hair-colouring products containing p-phenylenediamine. Contact Dermat..

[34] de Ávila R.I., Rodrigues T.L., Valadares M.C. (2020). Effects of hair dye ingredients on the oxidative stress response: Modulation of the mRNA expressions of NRF2, HO-1, and FOS in HaCaT keratinocytes. Contact Dermat..

[35] Moore A.I., Moreira A.S.P., Guerra I.M.S., Goracci L., Domingues P., Melo T., Domingues M.R., O’Boyle N.M. (2025). A lipidomic approach towards identifying the effects of fragrance hydroperoxides on keratinocytes. Contact Dermat..

[36] SCCP (Scientific Committee on Consumer Products) Opinion on Benzophenone-3, 16 December 2008. https://ec.europa.eu/health/ph_risk/committees/04_sccp/docs/sccp_o_159.pdf.

[37] Cosmetic Ingredient Review. Amended Safety Assessment of Benzophenones as Used in Cosmetics, 2021. https://cir-reports.cir-safety.org/cir-ingredient-status-report/?id=66345eff-e87a-ec11-8d21-000d3a991547.

[38] Oeda S., Hirota M., Nishida H., Ashikaga T., Sasa H., Aiba S., Tokura Y., Kouzuki H. (2016). Development of an in vitro photosensitization test based on changes of cell-surface thiols and amines as biomarkers: The photo-SH/NH2 test. J. Toxicol. Sci..

[39] Nishida H., Ohtake T., Ashikaga T., Hirota M., Onoue S., Seto Y., Tokura Y., Kouzuki H. (2021). In chemico sequential testing strategy for assessing the photoallegic potential. Toxicol. In Vitro.

[40] Onoue S., Ohtake H., Suzuki G. (2016). Comparative study on prediction performance of photosafety testing tools on photoallergens. Toxicol. In Vitro.

[41] Sukakul T., Svedman C. (2025). What is New in Contact Allergy to Cosmetics for Physicians, Cosmetologists, and Cosmetic Users?. Curr. Allergy Asthma Rep..

[42] Gupta M., Mahajan V.K., Mehta K.S., Chauhan P.S. (2015). Hair dye dermatitis and p-phenylenediamine contact sensitivity: A preliminary report. Indian Dermatol. Online J..

[43] Mukkanna K.S., Stone N.M., Ingram J.R. (2017). Para-phenylenediamine allergy: Current perspectives on diagnosis and management. J. Asthma Allergy.

[44] Serini S., Cassano R., Facchinetti E., Amendola G., Trombino S., Calviello G. (2019). Anti-Irritant and Anti-Inflammatory Effects of DHA Encapsulated in Resveratrol-Based Solid Lipid Nanoparticles in Human Keratinocytes. Nutrients.

[45] Fasano E., Serini S., Mondella N., Trombino S., Celleno L., Lanza P., Cittadini A., Calviello G. (2014). Antioxidant and anti-inflammatory effects of selected natural compounds contained in a dietary supplement on two human immortalized keratinocyte lines. Biomed. Res. Int..

[46] Jang H.Y., Kim G.B., Kim J.M., Kang S.Y., Youn H.J., Park J., Ro S.Y., Chung E.Y., Park K.H., Kim J.S. (2023). Fisetin Inhibits UVA-Induced Expression of MMP-1 and MMP-3 through the NOX/ROS/MAPK Pathway in Human Dermal Fibroblasts and Human Epidermal Keratinocytes. Int. J. Mol. Sci..

[47] Galbiati V., Papale A., Galli C.L., Marinovich M., Corsini E. (2014). Role of ROS and HMGB1 in contact allergen-induced IL-18 production in human keratinocytes. J. Investig. Dermatol..

[48] Kim H., Shin C.Y., Park C.H., Lee D.H., Lee S.H., Chung J.H. (2024). The pivotal role of osteopontin in UV-induced skin inflammation in a mouse model. Open Biol..

[49] Aubry L., Vallion R., Salman S., Damiens M.H., Ferret P.J., Kerdine-Römer S. (2023). Ethylhexadecyldimethylammonium bromide, a quaternary ammonium compound, controls inflammatory response through NRF2 pathway in a human immortalized keratinocyte cell line. Front. Toxicol..

[50] Yamaguchi H.L., Yamaguchi Y., Peeva E. (2023). Role of Innate Immunity in Allergic Contact Dermatitis: An Update. Int. J. Mol. Sci..

[51] Fitsiou E., Pulido T., Campisi J., Alimirah F., Demaria M. (2021). Cellular Senescence and the Senescence-Associated Secretory Phenotype as Drivers of Skin Photoaging. J. Investig. Dermatol..

[52] Feng C., Chen X., Yin X., Jiang Y., Zhao C. (2024). Matrix Metalloproteinases on Skin Photoaging. J. Cosmet. Dermatol..

[53] Wojciechowska M., Czajkowski R., Kowaliszyn B., Żbikowska-Gotz M., Bartuzi Z. (2015). Analysis of skin patch test results and metalloproteinase-2 levels in a patient with contact dermatitis. Postep. Dermatol. Alergol..

[54] Oxholm A., Oxholm P., Avnstorp C., Bendtzen K. (1991). Keratinocyte-expression of interleukin-6 but not of tumour necrosis factor-alpha is increased in the allergic and the irritant patch test reaction. Acta Derm. Venereol..

[55] Barker J.N. (1992). Role of keratinocytes in allergic contact dermatitis. Contact Dermat..

[56] European Comission Roadmap Towards Phasing out Animal Testing. https://single-market-economy.ec.europa.eu/sectors/chemicals/reach/roadmap-towards-phasing-out-animal-testing_en.

[57] OECD (2026). Test No. 498: In Vitro Phototoxicity—Reconstructed Human Epidermis Phototoxicity Test Method. OECD Guidelines for the Testing of Chemicals, Section 4.

