# Supplementary material for: Toward Animal-Free Phototoxicity Testing: A Keratinocyte-Based UVA Screening Assay to Distinguish Photoirritation from Photoallergy
Source: Toxics. 2026 Aug 16;14(8):726. doi: 10.3390/toxics14080726 (PMC13517369; doi:10.3390/toxics14080726)
Supplement: Supplementary file 1 [file toxics-14-00726-s001.zip › toxics-4461166-supplementary.pdf]

toxics

MDPI

Supplementary Materials

Toward Animal-Free Phototoxicity Testing: A Keratinocyte-Based UVA Screening Assay to Distinguish Photoirritation from Photoallergy

Adriana S. Maddaleno[1], Elisabet Teixidó[2], Laia Guardia-Escoté[2], Montserrat Mitjans[1,*] and Maria Pilar Vinardell[1,*]

[1] Physiology, Department of Biochemistry and Physiology, Universitat de Barcelona, 08028 Barcelona, Spain; adrianamaddaleno@ub.edu
[2] Toxicology, Department of Pharmacology, Toxicology and Therapeutic Chemistry, Universitat de Barcelona, 08028 Barcelona, Spain; eteixido1511@ub.edu (E.T.); laia.guardia@ub.edu (L.G.-E.)
* Correspondence: montsemitjans@ub.edu (M.M.); mpvinardellmh@ub.edu (M.P.V.)

1. *p*-phenylenediamine potential interferences

To evaluate the potential interferences of *p*-phenylenediamine on the MTT readings, PPD at a final concentration of 100 µg/mL in DMEM 5 % FBS and RPMI 5% FBS was introduced in a plate of 24 wells and exposed to 4 J/cm² or maintained in dark conditions. At the end of exposure, the plate was incubated at 37°C in a 5% carbon dioxide humidified incubator. After approximately 24 hours, the medium was extracted with a pipette and a solution of 0.5 mg/mL of MTT in free FBS DMEM or RPMI was added and incubated for 3 hours in the dark at 37°C in 5% CO_2. For control purposes, wells with medium without MTT and wells only with MTT solution were included in the plate. At each step, observation of color changes (Figure S1) was carried out.

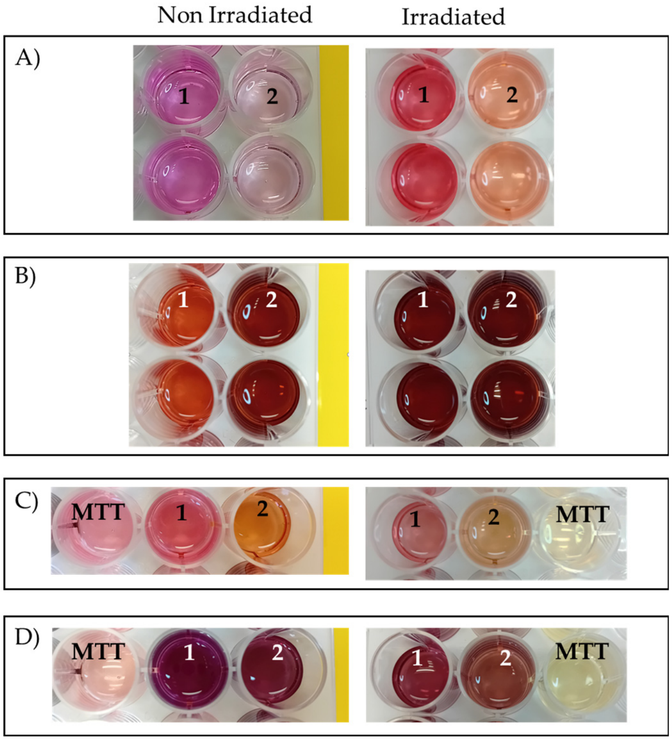

Figure S1. Interferences of PPD with MTT readings. A) Color of DMEM (1) and RPMI (2) 5% FBS containing 100 µg/mL of PPD in non-irradiated and irradiated conditions. B) Color changes of the same media with 100 µg/mL of PPD, non-irradiated or irradiated after 24 hours of incubation. C) Initial color of 0.5 mg/mL of MTT in free FBS DMEM (MTT) and with the remaining from the previous incubation. D) Final coloration after 3 hours of incubation.

As observed in Figure 1S, after irradiation with UVA light culture media became more brownish (a) and darkened after 24 hours (b). When the MTT solution is incubated, the few media with PPD that may remain can reduce MTT and the well contents became brown-purple (d), while wells with only MTT solution did not change the color. Although after UVA exposure, the media is removed before performing the MTT assay, this potential reduction due to the presence of PPD should be considered as a potential interference and overestimation of cell viability.

2. Intracellular IL-18 production.

Figure S2 shows the levels of intracellular IL-18 (pg/mg protein) obtained for HaCaT control cells in non-irradiated and irradiated conditions across the experiments performed. The intracellular IL-18 levels fluctuated considerably throughout the experiments, showing a minimum of 460 pg/mg, a maximum of 8,600 pg/mg, and a median of 2,800 pg/mg, approximately. The existence of such fluctuations had previously been described by other authors, indicating that cell density as a critical point and confluence must be avoided to maintain the discriminatory capacity of the cells (Galbiati et al., 2011; Galbiati et al., 2013). In this study, in addition to setting a cell density at 1×10^5 for all experiments, the cells were observed under a microscope prior to treatment to ensure they had not reached confluence. Nevertheless, other factors could account for the differences in IL-18 production, both inherent to the cells (such as cell passage), as well as other non-identified variables difficult to control.

Nevertheless, IL-18 production between non-irradiated and irradiated control cells is very similar, with inappreciable differences in most cases.

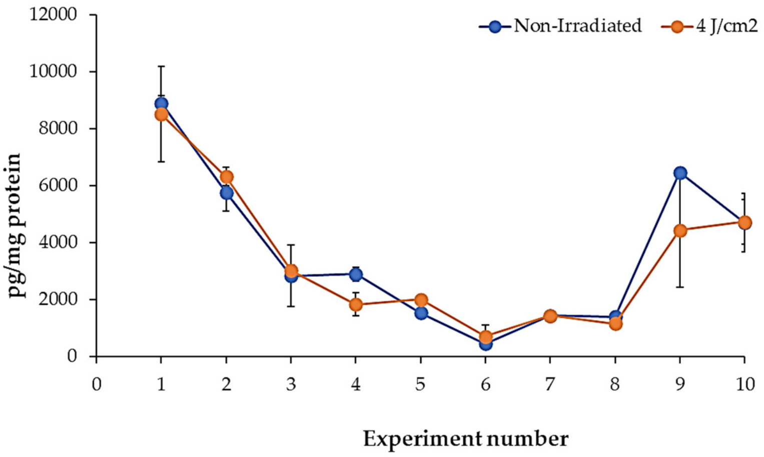

Figure S2. Intracellular IL-18 production by non-irradiated and irradiated at 4 J/cm^2 control cells across the experiments. Results are expressed as mean ± SD of 3 technical replicates.

3. Stimulation indexes in non-irradiated and irradiated conditions for IL-18

Table S1 presents the stimulation indexes calculated for the IL-18 production (pg/mg protein) for the chemicals studied at the three concentrations assayed.

Table S1. Stimulation indexes in non-irradiated and irradiated conditions for IL-18.

Chemical	µg/mL	NI*	I*	TSI*
CPZ	0.5	0.9 ± 0.1	0.9 ± 0.1	0.7 ± 0.1
	0.25	0.9 ± 0.1	0.9 ± 0.1	1.1 ± 0.2
	0.125	0.8 ± 0.1	0.5 ± 0.1$	1.1 ± 0.2
BZ	1.25	1.7 ± 0.4	2.5 ± 0.5$	2.1 ± 0.3
	2.5	2.0 ± 0.6	2.4 ± 0.4$	1.9 ± 0.3
	5	1.9 ± 0.6	1.4 ± 0.3	1.9 ± 0.4
8-MOP	0.005	0.9 ±0.1	0.9 ± 0.1	1.0 ± 0.1
	0.01	1.4 ± 0.3	0.8 ± 0.1	0.8 ± 0.1

	0.02	1.1 ± 0.1	0.8 ± 0.1	0.9 ± 0.0
PPD	10	0.9 ± 0.1	2.7 ± 0.7	2.9 ± 0.6[$]
	20	1.1 ± 0.3	2.5 ± 0.5	2.2 ± 0.3
	30	1.7 ± 0.6	2.0 ± 0.6	1.6 ± 0.4

[*] Results are expressed as mean ± SEM of 4 independent biological experiments. NI: stimulation index in non-irradiated conditions; I: stimulation index in 4 J/cm² conditions; TSI: total stimulation index. A one-way ANOVA followed by Dunnett's *post hoc* test was performed to evaluate statistically significant differences from controls. $^{\$}p < 0.05$; $^{\$\$}p < 0.01$

3. Chemiluminescence normalized values obtained with membrane arrays

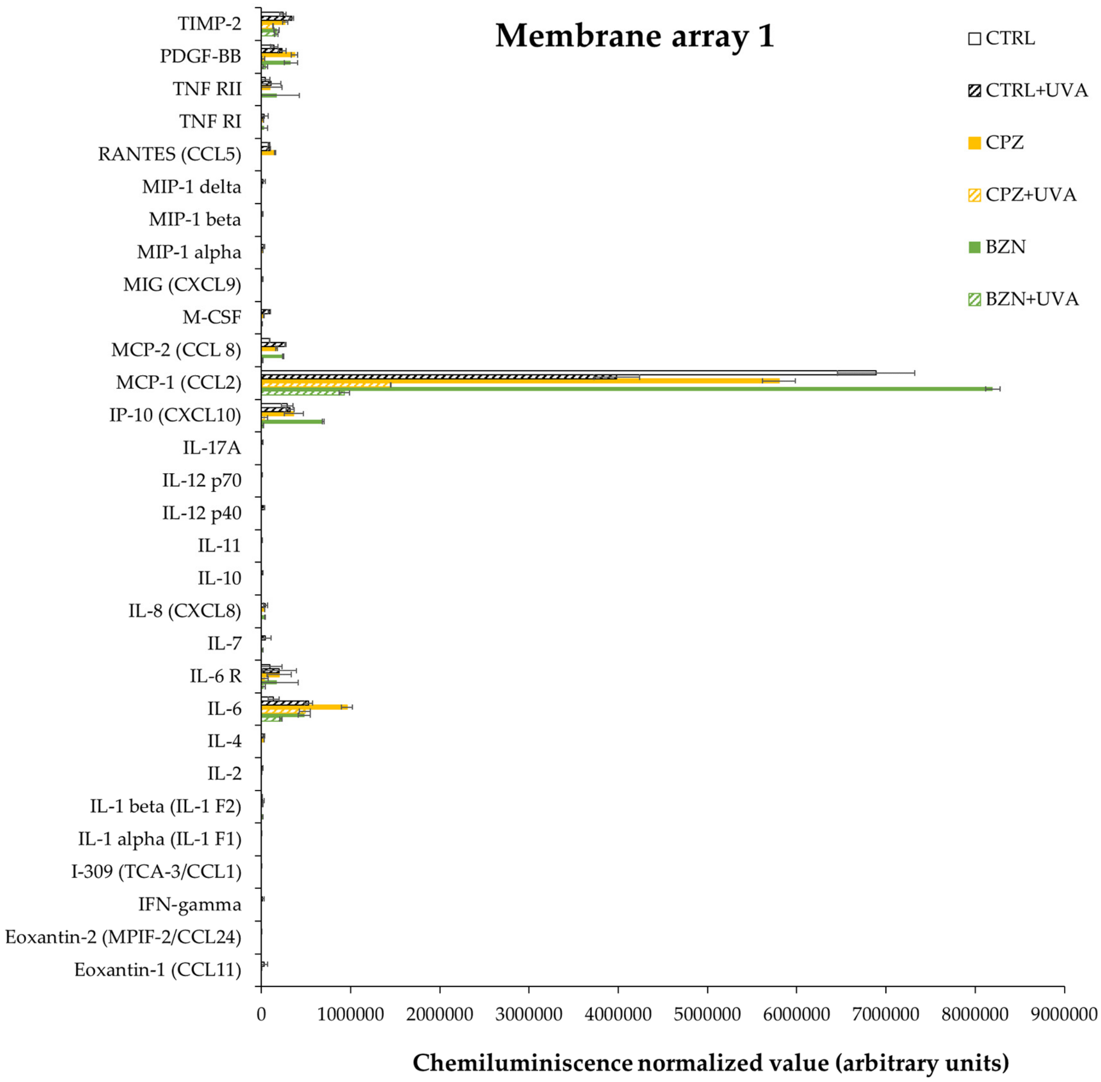

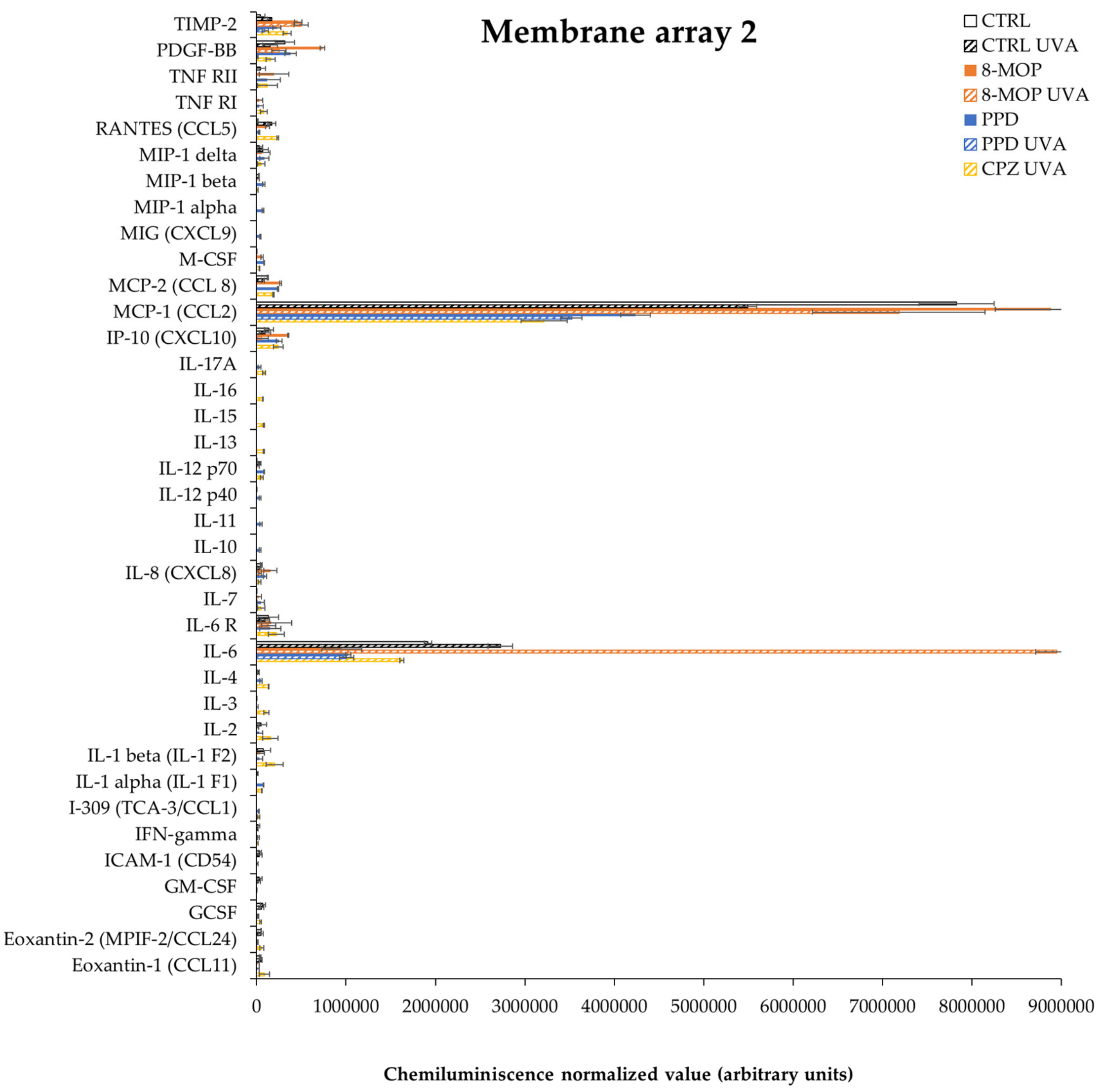

Figure S3. Chemiluminescence signals obtained with the cytokine inflammatory membrane arrays. Results are expressed as mean ± SD of two technical replicates for each cytokine detected. No signal was detected for GCSF, GM-CSF, ICAM-1 (CD54), IL-3, IL-13, IL-15, and IL-16 in array 1, and TGF beta1, TNF alpha and TNF beta in both arrays.

The relationships of these signals with respect to the untreated corresponding controls as well as the relationship between non-irradiated and irradiated (NI/I) are presented in Figure S4.

	Non-irradiated					4 J/cm^2				I/NI*			
**Cytokine**	**CPZ**	**BZ**	**8-MOP**	**PPD**	**UVA**	**CPZ**	**BZ**	**8-MOP**	**PPD**	**CPZ**	**BZ**	**8-MOP**	**PPD**
**Eoxantin-1 (CCL11)**	n.c	n.d	0.3	0.3	1.2	1.7	n.d	n.d	n.d	n.c	n.d	n.d	n.d
**Eoxantin-2 (MPIF-2/CCL24)**	n.d	n.d	n.d	0.1	0.9	1.2	n.d	n.d	n.d	n.c	n.d	n.d	n.d
**GCSF**	n.d	n.d	n.d	0.2	0.8	0.8	n.d	n.d	n.d	n.c	n.d	n.d	n.d
**GM-CSF**	n.d	n.d	n.d	0.0	0.7	n.d	n.d	n.d	n.d	n.d	n.d	n.d	n.d
**ICAM-1 (CD54)**	n.d	n.d	n.d	0.2	0.7	n.d	n.d	n.d	n.d	n.d	n.d	n.d	n.d
**IFN-gamma**	n.d	n.d	n.d	1.3	0.5	0.8	n.d	n.d	n.d	n.c	n.d	n.d	n.d
**I-309 (TCA-3/CCL1)**	n.d	n.d	n.d	n.c	n.d	n.d	n.d	n.d	n.d	n.c	n.d	n.d	n.d
**IL-1 alpha (IL-1 F1)**	n.d	n.d	n.d	10.5	n.d	n.d	n.d	n.d	n.d	n.c	n.d	n.d	n.d
**IL-1 beta (IL-1 F2)**	3.7	n.d	n.c	n.c	3.2	2.8	0.6	n.d	n.d	0.7	n.c	n.d	n.d
**IL-2**	n.c	n.d	n.c	n.c	n.c	3.4	n.d	n.d	n.d	n.c	n.d	n.d	n.d
**IL-3**	n.d	n.d	n.d	n.c	n.d	143.8	n.d	n.d	n.d	n.c	n.d	n.d	n.d
**IL-4**	n.c	n.d	n.d	n.c	n.c	7.6	n.d	n.d	n.d	n.c	n.d	n.d	n.d
**IL-6**	7.1	0.9	0.5	0.5	2.7	0.8	0.4	3.3	0.4	0.1	0.5	6.6	0.7
**IL-6 R**	2.1	1.8	1.3	1.2	1.4	1.2	0.1	1.4	n.d	0.6	0.0	1.1	n.d
**IL-7**	n.d	n.c	79.1	163.8	n.d	n.d	n.d	n.d	n.d	n.c	n.d	n.d	n.d
**IL-8 (CXCL8)**	n.c	n.c	2.9	1.8	0.5	1.2	n.d	1.4	n.d	n.c	n.d	0.5	n.d
**IL-10**	n.d	n.d	n.d	n.c	n.d	n.d	n.d	n.d	n.d	n.d	n.d	n.d	n.d
**IL-11**	n.d	n.d	n.d	n.c	n.d	n.d	n.d	n.d	n.d	n.d	n.d	n.d	n.d
**IL-12 p40**	n.d	n.d	n.d	n.c	n.c	n.d	n.d	n.d	n.d	n.c	n.d	n.d	n.d
**IL-12 p70**	n.d	n.d	40.3	292.8	116.8	1.8	n.d	n.d	n.d	n.c	n.d	n.d	n.d
**IL-13**	n.d	n.d	n.d	n.d	n.d	n.d	n.d	n.d	n.d	n.c	n.d	n.d	n.d
**IL-15**	n.d	n.d	n.d	n.d	n.d	n.d	n.d	n.d	n.d	n.c	n.d	n.d	n.d
**IL-16**	n.d	n.d	n.d	n.d	n.d	n.d	n.d	n.d	n.d	n.c	n.d	n.d	n.d
**IL-17A**	n.d	n.d	n.d	n.c	n.c	n.d	n.d	n.d	n.d	n.c	n.d	n.d	n.d
**IP-10 (CXCL10)**	1.3	2.1	2.6	1.8	0.9	1.4	0.0	0.6	n.d	1.1	0.0	0.2	n.d
**MCP-1 (CCL2)**	0.8	2.1	1.1	0.5	0.6	0.5	0.2	1.3	0.6	0.6	0.1	1.2	1.2
**MCP-2 (CCL 8)**	1.9	0.9	2.2	2.0	1.8	2.6	0.0	n.d	n.d	1.4	0.0	n.d	n.d
**M-CSF**	n.c	n.c	48.9	68.7	n.d	n.d	n.d	n.d	n.d	n.c	n.d	n.d	n.d
**MIG (CXCL9)**	n.d	n.d	n.d	n.c	n.d	n.d	n.d	n.d	n.d	n.d	n.d	n.d	n.d
**MIP-1 alpha**	n.c	n.d	n.d	n.c	n.d	n.d	n.d	n.d	n.d	n.d	n.d	n.d	n.d
**MIP-1 beta**	n.d	n.d	n.c	n.c	n.c	0.4	n.d	n.d	n.d	n.c	n.d	n.d	n.d
**MIP-1 delta**	n.c	n.d	2.3	3.2	2.5	0.8	n.d	n.d	n.d	n.c	n.d	n.d	n.d
**RANTES (CCL5)**	1.8	n.d	7.9	2.0	6.0	1.4	n.d	n.d	n.d	0.8	n.d	n.d	n.d
**TGF beta 1**	n.d	n.d	n.d	n.d	n.d	n.d	n.d	n.d	n.d	n.d	n.d	n.d	n.d
**TNF-alpha**	n.d	n.d	n.d	n.d	n.d	n.d	n.d	n.d	n.d	n.d	n.d	n.d	n.d
**TNF beta**	n.d	n.d	n.d	n.d	n.d	n.d	n.d	n.d	n.d	n.c	n.d	n.d	n.d
**TNF RI**	n.c	n.c	n.c	n.c	n.d	n.d	n.d	n.d	n.d	n.c	n.d	n.d	n.d
**TNF RII**	2.4	1.7	4.6	2.8	2.6	n.d	n.d	n.d	n.d	n.c	n.d	n.d	n.d
**PDGF-BB**	2.6	1.4	2.3	1.2	1.1	0.6	0.2	1.7	0.05	0.2	0.1	0.7	0.0
**TIMP-2**	1.1	0.5	11.2	5.5	2.7	1.2	0.5	3.0	0.6	1.1	1.0	0.3	0.1

Figure S4. Relationship of inflammatory cytokine chemiluminescence signals obtained after each treatment with the respective control. Non-irradiated samples and UVA controls were referred to as the non-irradiated control. Irradiated samples were referred to by UVA controls. I/NI: relationships of irradiated samples referred to the non-irradiated relationships. n. c. not calculated; n. d. not determined.

Data of chemiluminescence of the metalloproteinase membrane array are presented in Figure S5. In this case, we have detected all the proteins with the array under the different conditions assayed being the signals of MMP-1, MMP-10, TIMP-1 and TIMP-2 being the highest recorded. As observed, the chemiluminescence signal was more intense in the case of MMP-1, TIMP-1, and TIMP-2 in both assays performed.

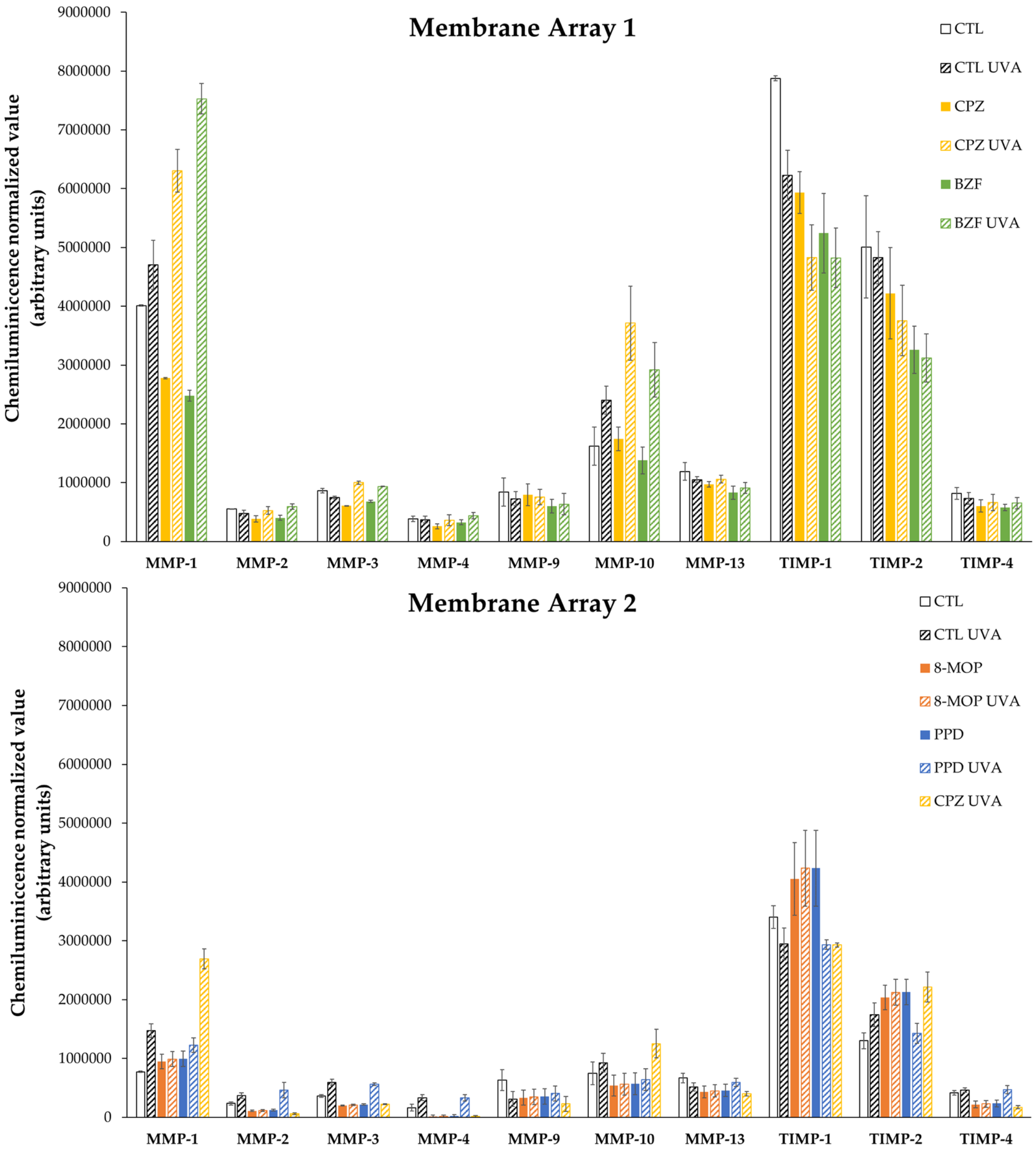

Figure S5 Chemiluminescence signals obtained with the metalloproteinase inflammatory membrane arrays. Results are expressed as mean ± SD of two technical replicates for each metalloproteinase detected.

The relationships of chemiluminescence obtained for each metalloproteinase in the different conditions studied were calculated relative to the signals detected for controls as shown in Figure S6.

	Non-irradiated					4 J/cm^2				I/NI*			
**MMPs**	**CPZ**	**BZ**	**8-MOP**	**PPD**	**UVA**	**CPZ**	**BZ**	**8-MOP**	**PPD**	**CPZ**	**BZ**	**8-MOP**	**PPD**
**MMP-1**	0.7	0.6	1.2	1.3	1.5	1.6	1.6	0.7	0.8	2.3	2.6	0.5	0.6
**MMP-2**	0.7	0.7	0.5	0.5	1.2	0.6	1.2	0.3	1.2	0.9	1.7	0.7	2.4
**MMP-3**	0.7	0.8	0.6	0.6	1.3	0.9	1.3	0.4	0.9	1.2	1.6	0.6	1.6
**MMP-4**	0.7	0.8	0.1	0.1	1.5	0.5	1.2	0.0	1.0	0.8	1.4	0.5	6.9
**MMP-9**	0.9	0.7	0.5	0.6	0.7	0.9	0.9	1.1	1.3	0.9	1.2	2.1	2.3
**MMP-10**	1.1	0.9	0.7	0.8	1.4	1.4	1.2	0.6	0.7	1.3	1.4	0.8	0.9
**MMP-13**	0.8	0.7	0.6	0.7	0.8	0.9	0.9	0.9	1.2	1.1	1.2	1.4	1.7
**TIMP-1**	0.8	0.7	1.2	1.2	0.8	0.9	0.8	1.4	1.0	1.2	1.2	1.2	0.8
**TIMP-2**	0.8	0.7	1.6	1.6	1.2	1.0	0.6	1.2	0.8	1.2	1.0	0.8	0.5
**TIMP-4**	0.7	0.7	0.5	0.6	1.0	0.6	0.9	0.5	1.0	0.9	1.3	0.9	1.8

Figure S6. Relationship of metalloproteinase chemiluminescence signals obtained after each treatment with the respective control. Non-irradiated samples and UVA controls were referred to as the non-irradiated control. Irradiated samples were referred to by UVA controls. I/NI: relationships of irradiated samples referred to the non-irradiated relationships.
